# Overcoming the blood–brain barrier for the therapy of malignant brain tumor: current status and prospects of drug delivery approaches

**DOI:** 10.1186/s12951-022-01610-7

**Published:** 2022-09-15

**Authors:** Ksenia Mitusova, Oleksii O. Peltek, Timofey E. Karpov, Albert R. Muslimov, Mikhail V. Zyuzin, Alexander S. Timin

**Affiliations:** 1 Peter The Great St. Petersburg Polytechnic University, Polytechnicheskaya 29, St. Petersburg, 195251 Russian Federation; 2 School of Physics and Engineering, ITMO University, Lomonosova 9, St. Petersburg, 191002 Russian Federation; 3 Sirius University of Science and Technology, Olympic Ave 1, Sirius, 354340 Russian Federation

**Keywords:** Brain tumor, Glioma, Glioblastoma, Blood–brain barrier, Invasive and non-invasive methods, Targeting vectors, Nanocarriers, Viral-like particles, Therapeutic drugs, Cell-based vehicles, Cell therapy

## Abstract

Besides the broad development of nanotechnological approaches for cancer diagnosis and therapy, currently, there is no significant progress in the treatment of different types of brain tumors. Therapeutic molecules crossing the blood–brain barrier (BBB) and reaching an appropriate targeting ability remain the key challenges. Many invasive and non-invasive methods, and various types of nanocarriers and their hybrids have been widely explored for brain tumor treatment. However, unfortunately, no crucial clinical translations were observed to date. In particular, chemotherapy and surgery remain the main methods for the therapy of brain tumors. Exploring the mechanisms of the BBB penetration in detail and investigating advanced drug delivery platforms are the key factors that could bring us closer to understanding the development of effective therapy against brain tumors. In this review, we discuss the most relevant aspects of the BBB penetration mechanisms, observing both invasive and non-invasive methods of drug delivery. We also review the recent progress in the development of functional drug delivery platforms, from viruses to cell-based vehicles, for brain tumor therapy. The destructive potential of chemotherapeutic drugs delivered to the brain tumor is also considered. This review then summarizes the existing challenges and future prospects in the use of drug delivery platforms for the treatment of brain tumors.

**Graphical Abstract:**

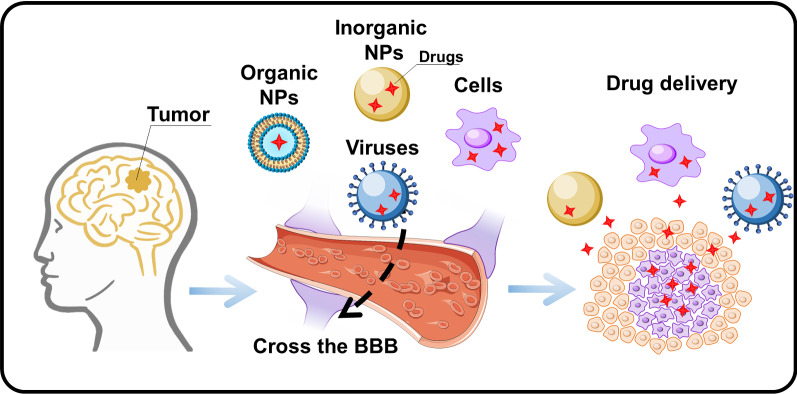

## Introduction

At the present moment, brain tumors hold the first place among all the primary central nervous system (CNS) tumors (85–90%). In 2020, more than 300,000 new cases of patients with brain tumors were diagnosed worldwide, out of which more than 250,000 deaths were registered [1]. Moreover, in 2020, the 5-year and 10-year survival rate for malignant brain tumors, which demonstrates the percentage of people who live at least 5 and 10 years after the tumor is diagnosed, were estimated to be around 36% and 31%, respectively [2]. For comparison, in 2000, these numbers were lower, 12% (5 years) and 9% (10 years). Despite the improvements in treatment opportunities for patients with malignant brain tumors, their survival rate is still low compared to other types of cancer (Table 1).

**Table 1:** The survival rate of different types of cancer

Type of cancer	Brain tumors(%)	Breast(%)	Melanoma(%)	Colon(%)	Multiple myeloma(%)
5-year survival rate	36	77–92	94	54	54
10-year survival rate	31	85	90	51	35
Ref	[2]	[3, 4]	[5, 6]	[7]	[8, 9]

In 2007, the World Health Organization (WHO) created the following histological classification of primary brain tumors:

- Neuroepithelial tumors, i.e. astrocytic tumors, oligodendroglial tumors, oligoastrocytoma tumors, ependymal tumors, glioma [10].
- Tumors of the meninges, i.e. meningioma, atypical meningioma, anaplastic meningioma [11].
- Tumors of cranial and paraspinal nerves, i.e. schwannoma, neurofibroma, perineurium, malignant peripheral nerve sheath tumor [12].
- Lymphomas and hematopoietic neoplasms, i.e. malignant lymphoma, plasmacytoma, granulocytic sarcoma [13].

Primary brain tumors arise from intracranial elements such as the cerebral hemispheres, the base of the skull, the hypothalamus, the basal ganglia, the thalamus, the brainstem, and the cerebellum. Globally, primary brain tumors are the 19th most common neoplasm, as reported by GLOBOCAN [14]. The global average incidence of primary brain tumors is 3.9 per 100,000 person-years. However, there is a significant difference between regions: the highest incidence rate is in Northern Europe (for example, Lithuania has an incidence of 8.0 and Norway of 5.4 per 100,000 person-years, respectively). After Northern Europe, Australia follows with a rate of 5.6, the US with 5.5, and Canada with an incidence of 5.3 per 100,000, respectively. In South America, the highest incidence is estimated to be found in Mexico, at 2.7 cases per 100,000 person-years [15]. Moreover, about 180,000 deaths from primary brain tumors are recorded annually in the world, which is 2.03% of all cancer deaths. The meningiomas (37%) and gliomas (25%) are the most widespread types of brain and other CNS tumors.

Meningiomas arise from the dural membranes of the brain. It is the most common intracranial tumor, accounting for 13–26% of all the primary intracranial tumors [16, 17]. Moreover, 10% of the population is unaware of the presence of meningioma due to the absence of symptoms [18].

Glioma is the most prevalent type of brain tumor [19]. More than 100,000 cases of diffuse gliomas are registered annually in the world. It has substantial mortality and morbidity [20]. The most lethal glioma, which accounts for 70–75% of all diagnoses of diffuse glioma, is Glioblastoma (GBM), with a median overall survival of 14–17 months (Table 2) [21–23]. On average, three people are diagnosed with GBM per 100,000 people [24]. GBM is distinguished by the following features: low patient survival, high detection rate among primary brain tumors and the lack of a wide range of therapeutic options, primarily due to the presence of the blood–brain barrier (BBB).

**Table 2:** Survival rating among primary brain tumors for patients of different ages

Type of cancer		Glioblastoma(%)	Low-grade (diffuse) astrocytoma(%)	Anaplastic astrocytoma(%)	Oligodendroglioma(%)	Anaplastic oligodendroglioma(%)	Ependymoma/anaplastic ependymoma(%)	Meningioma(%)
Age	20–44	22	73	58	90	76	92	84
	45–54	9	46	29	82	67	90	79
	55–64	6	26	15	69	45	87	74

BBB significantly complicates the treatment of primary brain tumors due to its low transmission capacity. In addition, the low BBB permeability seriously limits the potential application of the most prospective therapeutic drugs [25]. The BBB provides additional protection for neuronal tissues and enhances the destructive effect of cancer cells on the brain [26]. Therefore, there is a huge demand for developing effective approaches to deliver therapeutic agents through the BBB to treat GBM and other primary brain tumors [27, 28]

In this review, we have focused on the current challenges and prospects of overcoming the BBB challenge and reaching brain tumors, especially in the case of GBM and gliomas, using (i) clinically relevant drugs, (ii) different invasive and non-invasive methods, and (iii) organic/inorganic nanocarriers, viral-like particles (VLPs) and cell-based delivery systems to enhance the therapeutic efficacy against brain tumors. A major part of the review is devoted to the structural and functional features of the BBB, chemical modification of drug delivery systems, and the use of individual chemotherapeutic drugs to overcome the BBB.

## The structure of the BBB and its properties

The concept of a barrier between blood and the CNS arose at the end of the nineteenth century, in 1885, when Paul Ehrlich, a German doctor, immunologist and bacteriologist, discovered that the dye injected into the bloodstream of a rat had spread to all tissues and organs, excepting the brain [29]. He suggested the presence of some kind of barrier between blood and brain that serves as a filter for highly selective transfer of bioactive substances necessary for the metabolic activity of the brain and nervous system [30]. His conclusion was further confirmed by the later observations of his colleague Goldmann when he applied the same dye into the cerebrospinal fluid, and it did stain only the brain tissue [31]. This is how the concept of the BBB appeared.

The BBB plays a crucial role in the normal functioning of the CNS, and also controls the inflow and outflow of biological substances necessary for the brain [32]. The BBB is a biological dynamic membrane complex between the vessel lumen and the brain, which provides selective transport of molecules [33]. The barrier selectively absorbs ions, amino acids, glucose, and a range of nutrients to meet the nutritional and energy needs of the brain [34]. At the same time, the BBB prevents the penetration of various pathogens, metabolic products, and toxic compounds, preserving brain tissue from damage.

### The structure of the BBB

Generally, there are three main barriers in the brain [35]:

- *BBB* is formed by microvascular endothelial cells that line the brain capillaries [36]. These capillaries penetrate the brain and spinal cord. Due to the large surface area, the BBB is the largest interface between the blood and the brain. It protects the parenchyma of the brain from substances carried by blood, and also prevents the penetration of bioactive compounds into the brain and the CNS. The area of BBB varies from 12 to 18 m^2^, based on the average surface area of microvessels 150–200 cm^2^ per gram of tissue for an adult [37].
- *Blood–cerebrospinal fluid barrier* (BCSFB) is a barrier between blood and cerebrospinal fluid (CSF). It is formed by epithelial cells of the vascular plexus. The cells of the vascular plexus regulate the penetration of substances into the ventricles of the brain [38]. The reverse flow of the extracellular fluid of the brain is provided through the endothelium of the BBB capillaries [39].
- The *arachnoid barrier* consists of an avascular arachnoid epithelium [40]. It insignificantly contributes to the exchange between blood and the brain because of its limited surface area compared to other barriers [41].

**Fig. 1:**
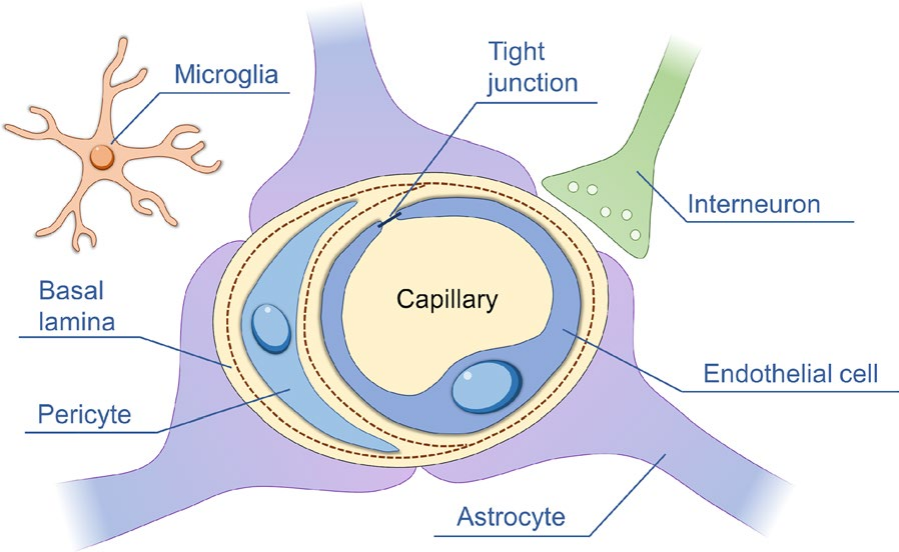
Schematic illustration of the BBB structure: capillary, endothelial cells, tight junction, basal lamina, pericytes, astrocytes, microglia, and interneuron

The BBB includes the endothelium of capillaries, which consists of its basal membrane and adjacent processes of gliocytes and pericytes (Fig. 1) [30, 42, 43]. By their structural organization, the capillaries of the brain are hemocapillaries with a continuous endothelial lining and a basal membrane. The endothelium plays an important role in the morphological structure of the BBB. Normal endothelial cells form a highly selective barrier for the passage of blood substances into the brain parenchyma [44].

The basal membrane is located under the endothelium and consists of pericytes, membrane organelles and ribosomes [45]. Pericytes maintain the tone of the basement membrane and participate in the motor regulation of capillaries [30, 46]. Astrocytes are located on the surface of blood vessels and are responsible for the transport of substances between capillaries and neurons[37]. Astroglia ensures the preservation of the BBB phenotype and promotes the regeneration of its endothelium [47]. Microglia provides immune defense in the brain. Aggregates of microglia cells form the brain’s own (internal) immune system. Microglia is one of the main morphological markers of the state of the brain in various pathologies and during experiments [45].

Thus, pericytes, embedded in the basal membrane of vessels and vascular cells of microglia, and astrocytes play a major role in the formation of dense BBB [48, 49]. Brain diseases usually result in a sharp increase in the permeability of the BBB [50], which is caused by mechanisms such as functional disruption of the integrity of interendothelial contacts, disruption of the barrier functions of endotheliocytes and glial cell membranes, and alterations of individual cellular elements forming the BBB [51].

### Physiological functions of BBB

#### Maintenance of ion homeostasis

The BBB is responsible for ion homeostasis of the brain microenvironment. Via ion channels, the BBB can regulate the concentration levels of potassium (K^+^), calcium (Ca^2+^) and magnesium (Mg^2+^) ions. For instance, the concentration of K^+^ in blood plasma is 1.8 times higher compared to the cerebrospinal and interstitial fluids [52, 53]. The homeostatic regulation through ion channels (K^+^, Ca^2+^ and Mg^2+^) provides normal function of the neural network [54].

#### Adjusting the level of neurotransmitters

Neurotransmitters are biologically active compounds carrying electrochemical signals from one neuron to other neurons through the synaptic space [55]. Neurotransmitters are essential for the normal function of the central and peripheral nervous systems. The BBB separates neurotransmitters and protects the brain from unexpected changes in the concentrations of neurotransmitters in blood plasma [31]. For instance, blood plasma contains neuroactive amino acids such as aspartate and glutamate, which can harm the brain tissues at a high concentration level. Due to the presence of the BBB, the concentration of aspartate and glutamate in the brain always remains at the required level,safe for the normal function of the brain [2, 36]

#### Regulation of the proteins transport from blood to the brain

The BBB participates in the formation of the CSF, which is produced from blood plasma by filtration in the vascular plexus. The filtration process occurs through the endothelium of the BBB capillaries. It allows the control over the level of transport proteins in the CSF, reducing their concentration in the CSF compared to that in blood plasma [56]. However, the BBB damage can result in the leakage of transport proteins to the CSF. As a consequence, transport proteins accumulate in the brain at a high concentration, which can further result in the dysfunction of CNS. For example, a high concentration of albumin, plasminogen, and prothrombin in the brain can initiate muscle cramp, glial activation or neuroinflammation [57].

#### Protecting the brain from neurotoxins

Many neurotoxic agents, including heavy metals, mefloquine, and food additives induce neurotoxicity and brain damage. These neurotoxins lead to brain injury with various side effects, such as neurodegeneration, reduced cognitive function, and increased psychiatric manifestations (i.e. depression, anxiety, sleep disturbances, and irritability). The BBB prevents these neurotoxic agents circulating in the blood from entering the brain. At the same time, the BBB regulates the transport of bioactive compounds into and out of the CNS (the so-called BBB permeability). However, a dysfunction of the BBB can lead to the leakage of these harmful blood components into the CNS, contributing to neurologic deficits [29, 37].

#### The mechanisms of transport through the BBB

**Fig. 2:**
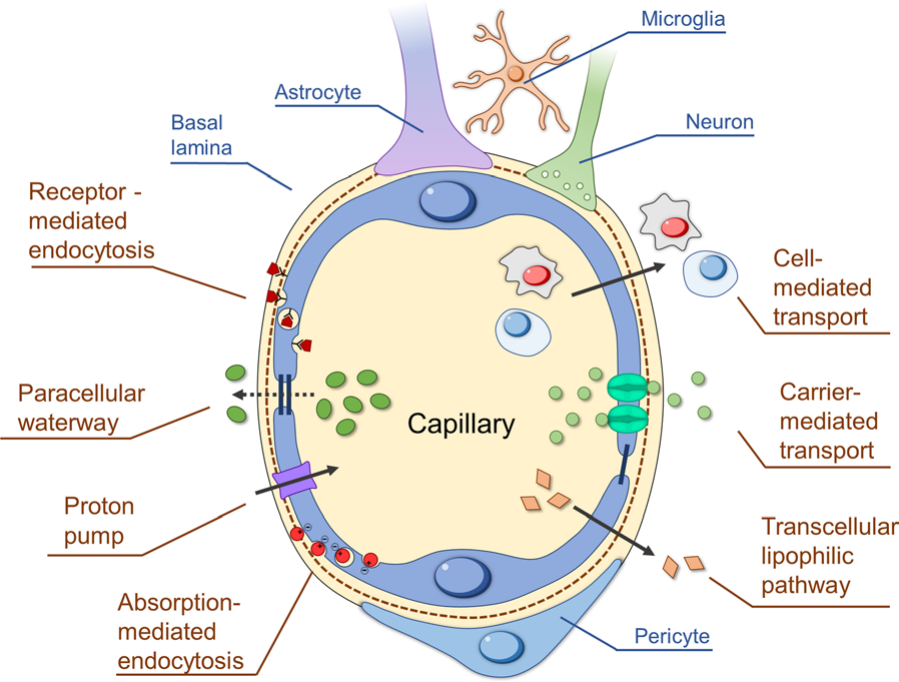
Mechanisms of transport through the BBB: diffusion (transcellular lipophilic pathway), carrier-mediated transport (CMT), receptor-mediated endocytosis (RME), absorption-mediated endocytosis (AME), proton pump, cell-mediated transport, and paracellular waterway

There are four main mechanisms that allow the molecules to cross through the BBB. They can be divided into passive transport (diffusion) and active transport (carrier-mediated transport, endocytosis, and cell-mediated transport) [58]. Passive transport occurs along a concentration gradient and does not depend on ATP energy, while active transport requires ATP hydrolysis and moves against a concentration gradient [59–61](Fig. 2).

#### Diffusion

Diffusion providing the transport of substances through the cell membrane can be divided into passive and facilitated diffusion [62]. Passive diffusion is the process of crossing the BBB driven by the concentration gradient of bioactive compounds between the blood and the extracellular fluid of the brain. A necessary condition for passive diffusion is the high lipophilicity of the substances. Thus, inorganic molecules such as O_2_, CO_2_, and H_2_O can quickly penetrate through the BBB. Numerous compounds that pass through the cell membrane via passive diffusion are pushed back to the vascular system by outflow pumps [37].

Facilitated diffusion is the transfer of substances through biological membranes mediated by specific carrier proteins, each of which is responsible for the transport of certain molecules or groups of related molecules. In the case of facilitated diffusion, substances are also transported in accordance with the concentration gradient, but the rate of this process is much higher than that of passive diffusion. The rate of facilitated diffusion has the property of saturation, which occurs when all the carrier molecules are occupied. Most of the essential nutrients, including amino acids, neurotransmitters, hormones, small peptides, etc., as well as small lipophilic molecules or therapeutic agents, enter the brain by facilitated diffusion [63]. This process includes a proton pump and a paracellular waterway. With the help of a proton pump, protons penetrate through the BBB, and the paracellular waterway is the transfer of water-soluble substances across the epithelium by passing through the intercellular space between cells [37].

Carrier-mediated transport (CMT) is an energy-dependent pathway based on the active transport of various bioactive compounds (usually small hydrophilic molecules). Biomolecules can be transported through the cell membrane via the symport (i.e. transport of two molecules in the same direction) and antiport (i.e. transport of two molecules in the opposite directions) mechanisms [64]. There are numerous proteins located in the BBB, which were shown to be responsible for its permeability. These proteins include, for example, glucose transporters (GLUT 1), large neutral amino acids (LAT1), monocarboxylic acids (MCT1), nucleosides (ENT 1–2, CNT1-2), and cationic amino acids (CAT1). There are several therapeutic molecules (i.e. L-DOPA, a-methyldopa, gabapentin, and melphalan) that can pass through the BBB via CMT. However, the efficiency of their delivery to the brain is limited [64, 65]

#### Endocytosis

Endocytosis is a multistep process in which bioactive compounds enter a cell through membrane invagination. Endocytosis regulates the interaction of cells with their microenvironment. Endocytosis is an energy-dependent transport mechanism and can be divided into three categories, including (i) pinocytosis, (ii) phagocytosis and (iii) mediated endocytosis [66, 67]. There are several stages of endocytosis. The first step is the membrane invagination, during which bioactive compounds are absorbed. Second, the cell membrane forms membrane-bound vesicles (the so-called endosomes) with bioactive compounds inside. Then, the formed endosomes transport the bioactive compounds towards the cell organelles via intracellular compartments. Finally, the bioactive compounds are released into cell cytoplasm by the destruction of endosomes. The latter phase of endosomes (endolysosomes) is involved in the degradation of bioactive compounds.

Pinocytosis is based on the cells absorbing fluids with dissolved molecules e.g., ions and proteins) in the size range of 0.07–2 μm. Next, phagocytosis is defined as the absorption of solid bioactive compounds by cells. A good example of phagocytosis is the absorption of various antigens (e.g., viruses, bacteria, and dead cells) by white blood cells (WBCs) [68]. There are several endocytosis mechanisms, including receptor-mediated endocytosis (RME) and absorption-mediated endocytosis (AME).

The RME relies on the receptor proteins located on the cell surface that can interact with specific ligands (drugs, hormones, growth factors, enzymes, and plasma proteins) in a complementary way. Transferrin, insulin, and leptin are good examples of molecules delivered to the brain through the RME mechanism [69]. Besides, there is a type of integral membrane (transmembrane) protein that can also transport specific ligands across the cell membrane. As an example, clathrin is the main transmembrane protein responsible for the formation of endosomes during the endocytosis process [70]. The main aspect in the delivery by clathrin-mediated endocytosis is the interaction of clathrin protein components with active sites of bioactive molecules. It further leads to the formation of clathrin-coated vesicles (early endosomes) for specific endocytosis [71].

The cell membrane of brain endothelium is negatively charged at physiological conditions. As a result, positively charged molecules can interact with negatively charged endothelial cells via electrostatic interaction, facilitating the AME mechanism. Contrary to the RME, the AME is not specific, but it has a greater binding capacity [72]. Overall, AME has the same transport efficiency through the BBB as the RME [73]. A variety of positively charged molecules can pass through the BBB via the AME, such as cationic proteins and positively charged polymers (PEI, chitosan). Surface modification of drug delivery systems with such molecules allows them to penetrate the BBB and get into the brain via the AME.

#### Cell-mediated transport

Macrophages, neutrophils, and monocytes can participate in the cell-mediated transport due to their high mobility, i.e. they can migrate across impermeable barriers and release the drug cargo at the sites of infection or tissue injury [74]. Their migration properties can also be used to deliver bioactive substances into the brain tumor. Therefore, these cells can act as “Trojan horses”, providing the transport of therapeutic drugs through the BBB [75, 76]. Among them, monocytes are the most appropriate cells for the transportation of bioactive compounds. Monocytes were proved to be able to transfer drugs to an inflamed area of the brain. However, cell-mediated transport has serious drawbacks, such as early release of drugs, unavailable targeted delivery, and poor drug loading capacity [74, 77]. Further, we will discuss the cell-based vehicles for delivery to brain tumors in detail.

## Clinically relevant chemotherapeutic drugs for the treatment of brain tumors

### Lipinski’s rule

Currently, chemotherapy is the main method used for brain tumor treatment. However, it has certain limitations and disadvantages. The therapeutic molecules that can penetrate through the BBB have similarities in their chemical structure. Lipinski et al. analyzed the properties of such molecules and formulated a set of rules that describe the molecules highly likely to be able to pass through the BBB [78]. The so-called Lipinski’s rule says that the therapeutic molecule is more likely to demonstrate a higher adsorption or permeation if it has less than 5 H-bonds, less than 10 H-acceptors and calculated Log P less than 5, and its molecular weight is no greater than 500 [79]. Development and testing of novel therapeutic molecules is an extremely long and expensive process, and Lipinski’s rule helps to accelerate it [80].

### Commercially available drugs

Depending on the type of brain tumor, various drugs are used for its treatment [81]. Nitrosoureas have the longest history in the treatment of malignant brain tumors. Carmustine and lomustine are two of the most extensively used nitrosourea compounds. These therapeutic agents are lipid-soluble and, therefore, capable of crossing the BBB. Lomustine is generally used in combined chemotherapy and is considered as the key factor in the PCV regimen (P: procarbazine, C: lomustine, V: vincristine). PCV used for high-grade gliomas has an overall survival of 6.7 months and progression-free survival of 3.6 months [82]. Carmustine is a non-specific alkylating agent that causes crosslinking of DNA and RNA, similarly to lomustine [83]. It demonstrates a higher toxicity and less effective treatment compared to other existing alternatives, thus resulting in less frequent use for high-grade glioma therapy [84].

Temozolomide (TMZ) was recently approved for use in the treatment of malignant gliomas as an oral chemotherapy agent. It is worth pointing out that TMZ confirms Lipinski's rule and demonstrates the highest BBB permeability among the drugs approved for glioma treatment. It is used in combination with surgery and/or radiation therapy, and the use of TMZ has significantly increased the median survival period. Nonetheless, subsequent treatment with TMZ is required, which fails in a number of cases due to the emergence of the TMZ resistance [85].

Carboplatin is a platinum-based chemotherapeutic agent extensively used in oncology. The cytostatic effect of carboplatin results from interference with DNA replication by inducing DNA cross-linkage. It demonstrates a lower BBB passage compared to the abovementioned temozolomide, and generally, a higher CNS toxicity. Therefore, it is usually prescribed to patients with a recurrent disease. Carboplatin demonstrates high clearance from the brain tissues, therefore, temporary BBB disruption during the treatment leads to higher efficiency of treatment [86, 87].

Irinotecan has shown limited efficacy due to low BBB penetration. Irinotecan interacts with topoisomerase I, which causes double-strand breaks. Similar to other chemotherapy agents, the response to this drug is not durable, and in the best scenarios, it is usually limited to several months. Therefore, development of new therapeutic drugs and formulations is required for novel and more effective approaches to brain tumor treatment [88].

Primary CNS lymphoma is another type of brain tumor (rare non-Hodgkin lymphoma) that is confined to the brain, cerebrospinal fluid and eyes [89]. Only several clinical trials have been performed, and there is still a lack of knowledge on the recurrence of this disease. Currently, there is no consensus regarding the optimal regimen for primary CNS lymphoma. However, high-dose methotrexate has proven to be effective in combination with other therapies, including surgery, chemo- and radiotherapy. Methotrexate is capable of penetrating the BBB, which made it much more effective compared to other chemotherapy agents used to treat non-CNS diffuse large B-cell lymphoma. Usually, high-dose methotrexate is used in combination with whole-brain radiotherapy, which has so far demonstrated a significant improvement in both overall response rate and prolonged progression-free survival from 3 to 18 months [90].

**Fig. 3:**
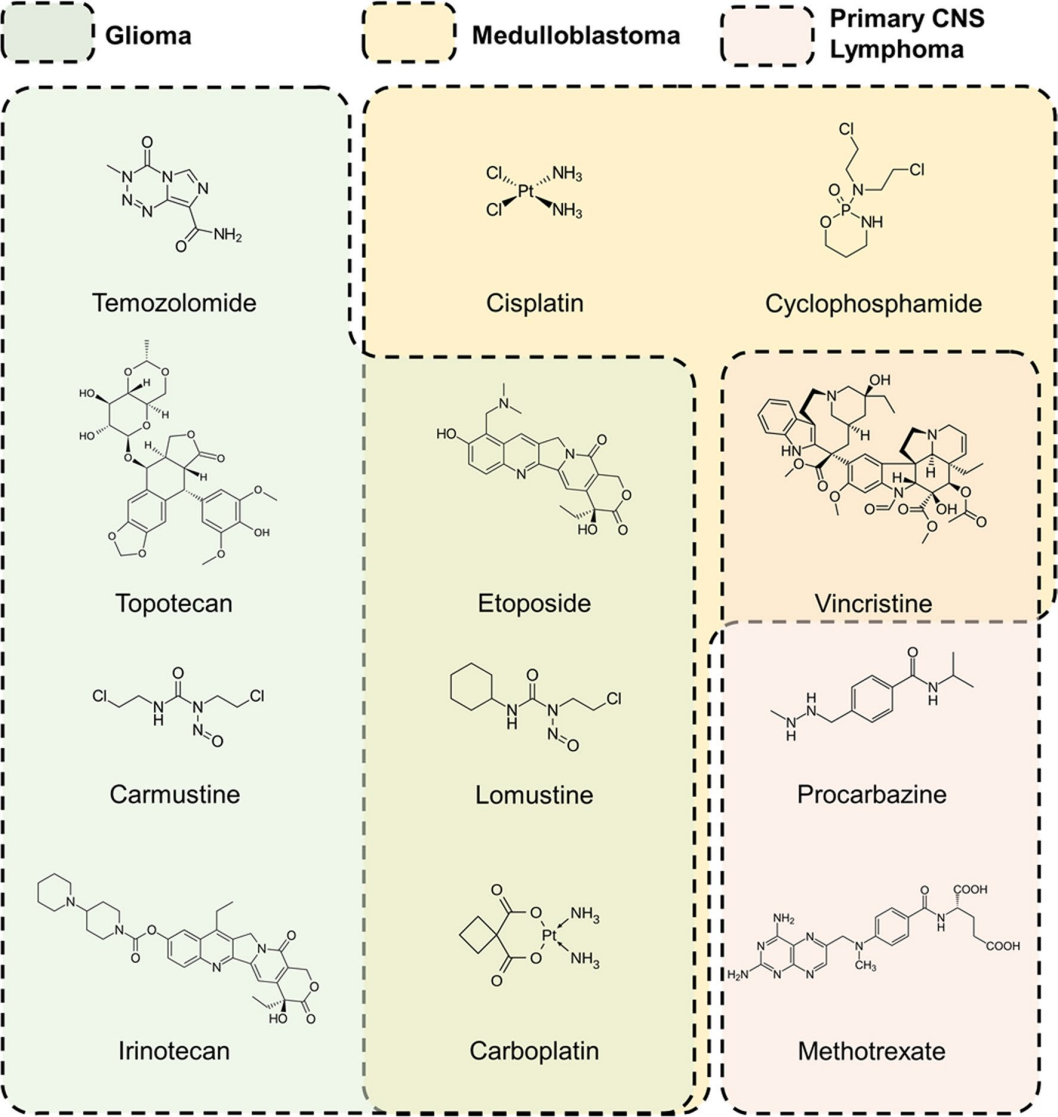
The commercially available drugs used for the therapy of different types of brain tumor, color coding: Glioma—green, Medulloblastoma—yellow, PCNSL—red. The cross-section means that the drug was used for both cancer treatments

The effectiveness of a chemotherapy agent for brain tumor treatment is mainly determined by the pharmacokinetics of the drugs. This means that a number of therapeutic molecules that are already used for chemotherapy could be utilized for brain tumor treatment if there was a way to improve their pharmacokinetics and transfer them through the BBB. For example, doxorubicin (DOX) is approved for GBM treatment, but it demonstrates low BBB permeability [91]. Another example is vincristine, a highly potent microtubule polymerization inhibitor extensively used in chemotherapy; however, similarly to DOX, it cannot penetrate the BBB and therefore requires a specific approach for effective delivery into the brain tumor [92]. The structures of commercially available drugs used for three types of brain tumor treatment (glioma, medulloblastoma and primary CNS lymphoma) are illustrated in Fig. 3.

## Invasive and non-invasive methods for overcoming the BBB

**Fig. 4:**
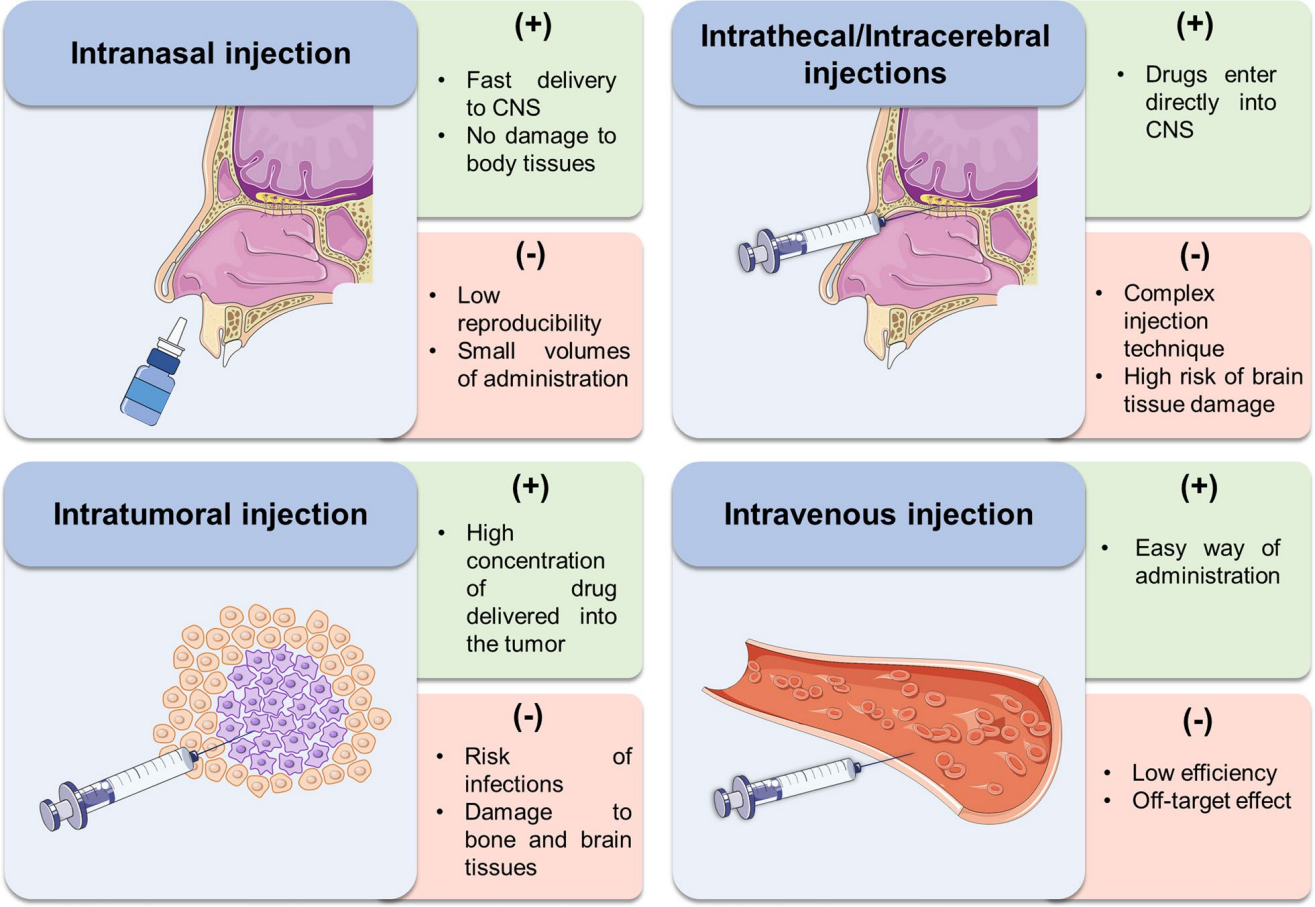
Schematic representation of intranasal, intrathecal/intracerebral, intratumoral and intravenous injections for the delivery of therapeutic molecules to the brain tumor

As previously discussed, many prospective antitumor drugs either cannot penetrate through the BBB or their delivery efficiency is very low. Therefore, more promising strategies for efficient drug delivery to the brain have been suggested [93]. These methods can be classified into two types: (i) invasive and (ii) non-invasive approaches. Invasive methods of drug delivery include intracerebroventricular (ICV), intrathecal, intracerebral and intratumoral injections [94]. ICV injections are mainly used for opioid therapy of the terminal stage of cancer to reduce the pain [95], and we will not consider this method of administration. Non-invasive methods include intravenous and intranasal administrations [96]. In this section, we mainly focus on intranasal, intrathecal, intracerebral, intratumoral and intravenous injections [97]. A schematic illustration of these methods with their advantages and disadvantages is presented in Fig. 4.

### Intrathecal and intracerebral injection

The intrathecal method involves the injection of drug directly into the epidural or subarachnoid space. The intrathecally injected molecules diffuse through the meningeal layers to reach the cerebrospinal fluid. Therefore, such a drug administration is expected to be more effective than intravenous injection. Many potential nanocarriers were investigated for intrathecal administration: for example, alginate/chitosan composite and maltosyl beta-cyclodextrin for improved delivery efficiency of bupivacaine [97, 98]. There are other examples of different nanocarriers administered by intrathecal injection, including polymer-based nanocomplexes [99–102] and inorganic nanocarriers [103]. However, the disadvantages of intrathecal injection are the risk of brain tissue damage, high changes in the intracranial pressure and low penetration of the drug through the BBB [100, 104, 105].

Intracerebral injection is the most direct method of drug delivery. In this method, intermittent bolus injections are locally administered into the brain, where the drugs further diffuse within the brain with minimal side effects [106]. This approach of drug delivery is commonly used for the treatment of primary brain tumors [107]. There are several preclinical studies of a therapeutic agent administered by intracerebral injection [108, 109]. Direct injections into the brain show good results in the treatment of brain tumors [110]. However, this method is very traumatic, and researchers are still to develop methods of treating brain tumors with the least harm to human health.

### Intratumoral injection

Intratumoral injection is another method for the delivery of therapeutic drugs to the brain tumor. Local drug delivery has been proposed to increase the concentration of the drug at the tumor site and reduce toxicity to the whole body [111]. For example, polymer structures consisting of liposomal DOX have received clinical approval for intracranial treatment of resectable GBM [112]. Direct infusion of fluid into the tumor has also been studied clinically. This method would allow the drug to remain in the tumor site for a long time, acting locally. However, complex implantation procedures, the risk of infections, local toxicity of drugs, and rapid removal of drugs from the brain parenchyma significantly limit the application of this method [113].

### Intravenous injection

An intravenous injection is the easiest way to administer a drug (the so-called arterial chemotherapy). Intravenous administration is associated with the use of drug delivery systems and occurs via CMT, RME, and AME (it will be discussed later). The advantages of the intravenous method include simple injection of the required dose of therapeutic drugs in the blood and variation of this dosage. Moreover, intravenous administration can be stopped if undesirable effects occur. However, this method has several disadvantages, mainly associated with the destructive mechanism of drug delivery through the BBB [114–116]. It can further lead to the side effects such as aphasia, hemiparesis, or even intracranial hernia [117]. The intravenous injection can also result in a non-specific accumulation of therapeutic drugs in other organs, i.e. the liver, spleen, and kidneys.

### Intranasal delivery

The intranasal route is an alternative method for the delivery of therapeutic drugs directly to the brain tissue. It first appeared in 1989 for the direct delivery of neurotrophic factors to the brain [118]. According to the literature, the intranasal delivery of the therapeutic drugs is achieved through the trigeminal and the olfactory nerves, directly transporting the drugs into the CSF while bypassing the BBB [119]. Compared with traditional intravenous administration, the intranasal delivery has several advantages, including simplicity, safety, convenience, and painlessness [120]. Further, it does not require aseptic techniques. The main strength of intranasal delivery is the rapid absorption of drugs in the nasal cavity. At the same time, there are some drawbacks associated with low reproducibility of this method [121, 122]. In general, individual drugs are used for intranasal delivery. Currently, a great interest has been aroused in the application of nanocarriers to deliver the drugs via the intranasal pathway [123]. However, the nasal architecture limits the delivery of nanocarriers along the intranasal route. Moreover, a special device is needed for the intranasal administration of a required dose of nanocarriers formulation. Various devices can be used for this purpose, i.e. from a simple nasal spray to a more complex instrument such as an electronic atomizer [124].

## The use of targeting vectors for overcoming the BBB

Therapeutic molecules or nanocarriers with a relatively large size can be delivered into the brain via special receptors expressed by the endothelial cells of the BBB [125]. On the surface of these cells, a large number of different receptors (transferrin receptor, insulin receptor, and nicotinic acetylcholine receptor) and transporters of metabolic nutrients are expressed. Therefore, the modification of drugs or nanocarriers with ligands that are complementary to these special receptors can provide the receptor-mediated mechanism for overcoming the BBB, allowing them to penetrate the brain tumors [126, 127]. Many targeting ligands, e.g. monoclonal antibodies (MAbs) and short peptides against receptors expressed on the BBB were used for surface conjugation of nanocarriers or chemical modification of antitumor drugs to achieve brain targeting [126]. Individual targeting ligands (MAbs, peptides, glucose, L-DOPA) can also serve as monotherapeutic agents themselves, which enables them to overcome the BBB.

Another way of penetration is via specific transporters on the surface of the BBB. Some low molecular weight compounds are able to penetrate the BBB with the help of specific transporters, for example, the large neutral amino acid transporter 1 (LAT1) for L-DOPA [128–130 ] or the efflux transporter P-glycoprotein (P-gp) for lipophilic drugs [131, 132].

In the following sections, the main targeting vectors are considered for monotherapy and chemical modification of therapeutic drugs or nanocarriers that can provide receptor- and carrier-mediated delivery of drugs into brain tumors.

### Glucose

**Fig. 5:**
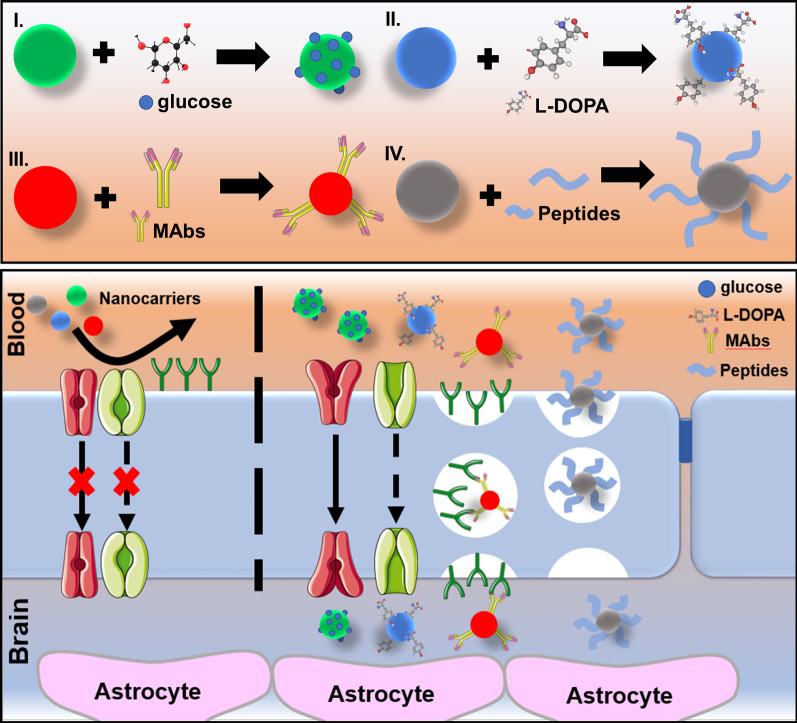
Schematic illustration demonstrating the surface modification of nanocarriers with targeting vectors (glucose, L-DOPA, MAbs, and peptides) and the process of the BBB penetration

As previously mentioned, RME is the main mechanism for the penetration of compounds through the BBB [133]. The d-glucose transport protein (GLUT) (Fig. 5) produces a particularly high concentration in the microvessels of the brain [134]. The concentration of GLUT receptors is almost 100 times higher than that of transferrin receptors, which are actively used as a specific ligand for targeted delivery of drugs or nanocarriers to the brain tissue. The glucose transported across the BBB with the help of GLUT provides almost all of the energy required for normal brain function. A detailed study of GLUT suggests that this receptor can be an effective target for the delivery of glucose-modified drugs to the brain through the BBB [135]. Moreover, GLUT has been shown to be overexpressed in brain tumor cells. Therefore, conjugation of the anticancer drugs with glucose imparts additional tumor tropism. The therapeutic molecule labeled with glucose penetrates through BBB due to the GLUT receptors. Then, the cancer cells overexpressing GLUT provide the targeted accumulation of drugs in the tumor [136].

It was shown that glucose-modified liposomes can penetrate through the BBB several times more effectively than without glucose conjugation [137]. Later, Li et al. demonstrated that glucose-modified liposomes loaded with antitumor drugs can effectively penetrate the brain and release the drug, producing a high local concentration in the region of brain tumors [138]. Many works have shown the efficiency of glucose as a targeting ligand for the delivery of drugs or nanocarriers into the brain tumors [133, 137, 139–142]. Polt et al. have shown that glucose-modified peptides were successfully used for the BBB penetration. Moreover, glucose improves the penetration of peptides into the brain with the help of GLUT, and glucosylation reduces the lipophilicity of peptides, including the mechanism of transport of lipophilic substances [140]. Fu et al. have synthesized unique glucose with RGD peptide (glucose-RGD) derivative that can target glioma [143]. The glucose-RGD was further used for the surface modification of liposomes loaded with an antitumor agent. This modification has increased the degree of penetration of liposomes through the BBB [141]. Tamba and others modified inorganic nanocarriers with poly(ethylene glycol) glucose methyl ester amine. Their in vivo results have shown that the modified nanocarriers were able to penetrate through the BBB into the brain [144].

Cancer cells consume much more glucose than healthy cells. This process is known as the Warburg effect. It is caused by tumor cell hypoxia, genetic mutations, and mitochondrial abnormalities in proliferating cancer cells [145]. The rapid and aggressive proliferation characteristic of tumor cells is itself a very energy-consuming process, so it is quite obvious that tumor cells consume much more glucose [146]. The Warburg effect has a significant impact on tumor cells, including their stimulation growth, providing ATP consumption under hypoxic conditions, regenerating endogenous antioxidants, acidifying the microenvironment, and producing carbon to increase biomass [147]. These changes can induce unregulated glucose fermentation pathways for the energy supply and growth of cancerous cells. This process was investigated using positron emission tomography (PET) [148]. Lieberman and others demonstrated that 4-18F-(2S,4R)-fluoroglutamine (^18^F-FGln) as an analogue of 18F-fluorodeoxyglucose (^18^F-FDG) can accumulate in glioma and GBM with great efficiency. The results of PET imaging clearly confirmed the accumulation of ^18^F-FGln in the GBM (rat model) [149].

### L-DOPA

L-DOPA (L-3,4-dihydroxyphenylalanine) is a precursor to dopamine that passes through the BBB due to large neutral amino acids LAT1 (Fig. 5). The ability of L-DOPA to effectively cross the BBB without causing toxic effects has been extensively investigated [150–152]. Dopamine, which is a hydrophilic, water-soluble neurotransmitter, is unable to penetrate the BBB, while its precursor, L-DOPA, can overcome the BBB with great efficiency [153, 154].

In the case of patients with advanced GBM, dopamine replacement strategy has been adopted as a gold standard since the late 1960s through the use of a dopamine precursor known as Levodopa or L-DOPA [155]. L-DOPA can be used in monotherapy, or in combination with antitumor drugs, increasing the efficiency of drug penetration through the BBB.

Bhunia et al. have successfully used Amphi-DOPA liposomes to improve the delivery efficiency of chemotherapeutic drugs through the BBB directly to GBM (in vivo model) [156]. German scientists have successfully implemented L-DOPA modified with radioactive fluorine-18 (^18^F-DOPA) for a more accurate diagnosis of patients with GBM. The developed structure of ^18^F-DOPA allows to increase the level of diagnostics by 39%, and therapy by 17% [157]. A study by Capuani and colleagues demonstrated a significant increase in the uptake of boronophenylalanine in brain tumors with the use of L-DOPA. Preloaded L-DOPA passed through the BBB and accumulated in glioma cells, and then it induced a significant increase in the efficiency of cancer cell elimination. No side effects were recorded in healthy tissues of the body [158]. Gonzalez-Carter developed inorganic nanocarriers modified with L-DOPA (L-DOPA-AuNF). The scientists were able to demonstrate that L-DOPA-AuNF crosses the BBB much more efficiently than non-modified nanocarriers and without any serious side effects [159].

### L-carnitine

L-carnitine is a natural compound found in almost all the tissues of the human body, including the brain [160]. The main function of L-carnitine is the transport of activated long-chain fatty acids (long-chain fatty acyl-CoA) into the mitochondria for the β-oxidation process [160, 161]. Human brain tissues contain free L-carnitine and its acylated derivatives with carbon chains of various lengths, including acetylated and palmitoylated derivatives. All these derivatives can transport therapeutic compounds through the BBB [161]. Over the past 5 years, the interest in the therapeutic potential of l-carnitine and acetyl-l-carnitine (ALCAR) for the transport of compounds across the BBB and for neuroprotection has significantly increased [162–167].

Nałęcz et al. have studied the organic cation transporter (OCTN2) and the solute transporter (SLC22A5). Both carriers are able to interact with L-carnitine and deliver it through the BBB. On the one hand, it allows tumor cells to receive an additional source of energy in the form of glucose and thereby grow and proliferate. On the other hand, it was proved that SLC22A5 can also provide targeted delivery of chemotherapy drugs combined with carnitine to brain cancer cells [168]. Taking advantage of the specific expression of Na^+^-OCTN2 on both brain capillary endothelial cells and glioma cells, l-carnitine was used for the surface modification of nanocarriers. For instance, Kou et al. have developed polymeric nanocarriers conjugated with l-carnitine. During in vitro studies, it was shown that the conjugation of l-carnitine significantly improved the uptake of nanocarriers by endothelial cells of the BBB and glioma cells. Moreover, in vivo studies demonstrated a high accumulation of l-carnitine modified nanocarriers in the brain, as was confirmed by fluorescent imaging assays. Finally, the obtained l-carnitine-modified nanocarriers were loaded with paclitaxel as an antitumor drug. The drug-loaded carriers showed an improved anti-glioma efficiency compared to non-modified nanocarriers [169]. Mingorance and colleagues have shown that acetyl-l-carnitine has better BBB penetration than regular l-carnitine and can additionally protect mitochondria from the oxidative process, and provide an overall neuroprotection for healthy cells of the nervous system [170]. Yamada et al. have studied the effects of carnitine on GBM cells. According to the obtained data, carnitine had an antioxidant effect in healthy cells while inducing the process of apoptosis in GBM cells [171, 172].

### Monoclonal antibodies (MAbs)

Several monoclonal antibodies (MAbs) specific to receptors on the surface of the BBB demonstrated the ability to increase the penetration efficiency of therapeutic molecules across the BBB. MAbs can cross the BBB, using receptor- and transport-mediated mechanisms [173–176]. First, in 1995, Pardridge et al. developed MAb83-7 and MAb83-14, which are specific to the human insulin receptor (HIR) [177]. MAb83-7 and MAb83-14 were able to bind to different epitopes of the active site of the insulin receptor, which contributed to a high rate of penetration through the BBB. The researchers examined the ability of MAbs to serve as drug carriers [178]. The biotnyl[^125^I]-Aβ 1–40 (amyloid beta) has been conjugated to MAbs specific to the HIR. The developed conjugate showed a high degree of penetration through the BBB, while Aβ 1–40 was unable to cross the barrier [179]. The enzyme iduronidase (IDUA) is often used as a therapeutic agent, but this enzyme cannot cross the BBB. IDUA conjugated to HIR-MAbs is able to provide enzyme replacement therapy by increasing the BBB penetration [180]. The MAb OX26 is a ligand for the human transferrin receptor (TfR) (Fig. 5). The experiments with laboratory mice have shown that OX26 MAb can be used as a drug delivery platform for crossing the BBB [181–183].

Bispecific antibodies (bsAbs) can be considered as a new generation of biomolecules with 2 different binding sites [184]. The bsAbs have a great potential of enhancing the BBB penetration. They have binding sites for TfR and β-site amyloid precursor protein cleaving enzyme 1 (BACE1). The bsAbs have a dual effect due to the binding affinity for TfR. If binding occurred with a low affinity for TfR, the level of BACE1 penetration through the BBB is increased [184, 185]. Low-density lipoprotein receptor-related protein 1 (LRP1) (Fig. 5) is responsible for the transport of bioactive compounds across the BBB, and therefore can be used as a promising target for MAbs [186, 187].

The study by Wikstrand et al. evaluated the use of boronated MAbs in boron-neutron capture therapy (BNCT) of glioma tumor (GBM rat model) [188]. Lampson has focused on the mechanisms of BBB penetration of several MAbs, including bevacizumab, rituximab, and trastuzumab for the treatment of GBM and primary central nervous system lymphoma (PCNSL). The obtained data indicated that overall survival tended to increase, which had a positive effect on the quality and time of life of patients with brain tumors [189]. This study also demonstrated that MAbs can be used not only as drug delivery platforms that increase the penetration of drugs through the BBB but also as individual therapeutic agents [189]. Later, Han et al. designed and synthesized MAb nanocapsules that contain acetylcholine and choline analogues for effective brain tumor suppression (orthotopic-glioma mice model) [173]. Galstyan et al. demonstrated trans-BBB delivery of nanoscale immunoconjugates, consisting of natural biopolymer scaffolds and MAbs inhibitors of α-CTLA-4 or α-PD-1 for activating both systemic and local brain tumor immune response [190]. Gan et al. summarized and analyzed clinical data on specific antibody-active substance conjugates that can cross the BBB [191]. Brain-derived neurotrophic factor (BDNF) is commonly used as a neuroprotective agent to prevent neuronal death after brain injury or development of brain tumors [192]. The conjugation of the BDNF with OX26 MAbs allows its transport into the brain via the BBB transferrin receptor transcytosis [193]. Thus, the complex of the drug and MAbs has a targeted action, the ability to penetrate the BBB, and a therapeutic effect on the necessary elements of the brain: damaged neurons and tumor cells [194].

### Peptides

Peptides can penetrate through the BBB with the help of special transporters [195], receptors [196], or using the lipophilicity of substances [197]. p-glycoprotein (P-gp) can restrict the entry to the brain for many therapeutic drugs. However, the co-administration of chemotherapeutic drugs with P-gp modulators can inhibit the influence of P-gp, increasing the brain clearance. For example, the anticancer drug such as DOX was conjugated with D-penetrin and SynB1 peptides as P-gp inhibitors to cross the DOX through the BBB [198]. A sixfold increase in the efficiency of drug penetration through the BBB was demonstrated compared to free DOX. Cell-penetrating peptides also showed their ability to enhance the penetration of bioactive compounds through the BBB [199, 200]. Insulin-like growth factor 2 (IGF2) is a peptide that can transport biomolecules from blood to the brain using RME because it has a high affinity to IGF receptors at the human BBB. However, the high binding affinity of IGF2 (> 99%) in the blood significantly limits the application of this peptide for brain tumor therapy [201].

Kumar et al. have written a comprehensive review, observing the peptides that can be used for surface modification of nanocarriers to extend the ability to target glioma tumors [202]. In that review, the authors discussed the prospects of peptide-decorated nanocarriers as a drug delivery vehicle for the controlled release of chemotherapeutic agents in a targeted manner. In the experimental work by Yao et al. a novel gene vector was created, based on dendrograft poly-l-lysines and polyethylene glycol (PEG) conjugated to a cell-penetrating peptide. Due to this peptide, the degree of penetration of the entire structure through the BBB significantly increased [203]. In 2019 Chen et al. designed a library for in vivo screening of peptides that can cross the BBB and bind to LRP1 [204].According to the analysis, a specific peptide that contributed to the passage of phages into the brain was identified. This peptide targeted and accumulated in brain tumors using the U87 glioma mice model. Sánchez-Navarro and colleagues have written an outstanding review on the use of a new generation of peptides for overcoming the BBB [205]. This type of peptide can transport active substances across the BBB, it has low systemic toxicity, and can be easily modified with therapeutic drugs. Laksitorini et al. demonstrated that cyclic-ADT peptides (ADTC1, ADTC5, and ADTC6) promote increased transport of marker molecules, such as 14C-mannitol, to the brain via the BBB [206]. ADTC5 doubled 14C-mannitol delivery to the rat brain. In addition, the ADTC5 peptide increased in vivo delivery of Gd-DTPA to the brain of mice when administered intravenously. Thus, ADTC5 can radically improve the delivery of diagnostic and therapeutic agents to the brain and increase the effectiveness of primary tumor therapy [207]. A conjugate of paclitaxel and angiopep-2, named ANG-1005, was synthesized by Prof. Régina's team [208]. It was previously reported that angiopep-2 can be used as a drug carrier for the BBB penetration. The angiopep-2 is a specific ligand for LRP1, which was detected in GBM and different brain metastases. ANG-1005 passed two parallel phase 1–2 clinical trials and demonstrated high accumulation in the GBM compared to individual paclitaxel [209].

## Drug delivery platforms

**Fig. 6:**
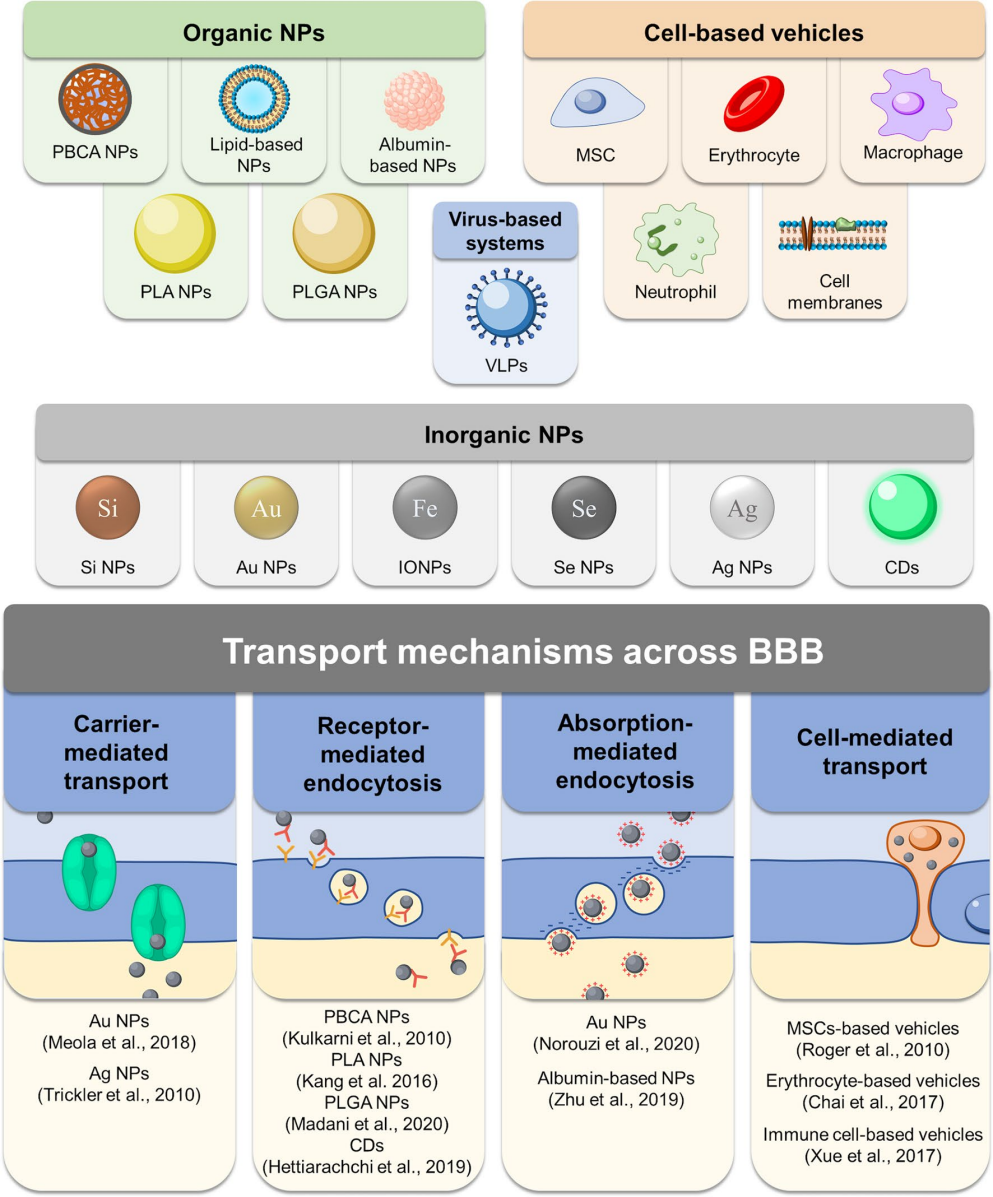
Schematic illustration depicting various drug delivery platforms (organic and inorganic NPs, VLPs, and cell-based vehicles) used for overcoming the BBB

Drug delivery platforms have many notable features compared to individual therapeutic drugs [209]. Their main advantages are the reduction of off-target effects and promotion of site-specific accumulation in the brain tumor, minimizing the systemic toxicity from therapeutic drugs [210]. The unique properties of the developed drug delivery vehicles can additionally provide in vivo tracking visualization of encapsulated drugs. To date, many materials, biological components, and therapeutic cells have been considered for the fabrication of these drug delivery platforms. They were classified into viral-like particles (VLPs), organic/inorganic nanoparticles (NPs), and cell-based delivery systems. Viral and cell-based delivery systems can be considered as separate categories of delivery systems extensively used for brain tumor therapy [211, 212] In the following chapters, we will discuss and summarize the data on various delivery systems for tumor brain therapy. The main structures of drug delivery systems with the corresponding typical mechanisms of BBB penetration are presented in Fig. 6.

### Virus-like particles (VLPs)

Virus-like particles (VLPs) form an immunogenic platform for the development of effective therapeutic anti-cancer vaccines against brain tumors (GBM, gliomas, etc.) [213–215]. VLPs can be used independently as a cancer vaccine [216] and as a carrier for the delivery of therapeutic compounds [217]. Rabies virus (RV) is often applied for trans-neural tracking [218]; on its basis, vaccines against cancer, in particular, gliomas, have been developed [219]. An RV-based vaccine (RV-V) can cause an overall increase in the immune activity that can be used against cancer. The RV envelope protein, glycoprotein (RVG), forms spikes that protrude from the viral envelope. Lymphocytes are activated in the presence of RV antigens, resulting in the production of antibodies against GBM cells [220, 221] Despite methodological problems, there are studies that showed increased survival in the patients with GBM that received RV-V therapy [220].

In 2009, the scientific group of Prof. Filipov reported clinical results of RV-V therapy against GBM [222]. Twenty patients with GBM were treated with RV-V, deferoxamine, and D-penicillamine. The vaccines were started shortly after optimal radiation therapy; and in 6 cases, treatment was initiated during the period of neurological deterioration. The patients that received chemotherapy were treated with vaccines for several weeks without chemotherapy. The median postoperative survival of the treated patients was 28 months. Nine people from twenty patients were alive, and five of them were in good condition. In 2013, a research group from Thomas Jefferson University reported that RV-V therapy significantly enhances the survival rate of mice with intracranial glial tumors GL261. The increase in survival rate was associated with delayed tumor growth and an increase in markers of T and B cells and IFNy in CNS tissue [220, 223]. Thus, the RV-V is a promising therapy against cancer, in particular GBM, increasing the effectiveness of existing immunotherapy methods.

RV-based VLPs can also be used as a delivery system for the efficient transport of therapeutic drugs to brain tumors [217]. We should note that the envelope protein and structural morphology of VLPs play an important role in overcoming the BBB and other physiological barriers [224]. The corresponding VLPs design was shown to provide a better biological distribution and a higher absorption efficiency by cancer cells than organic and inorganic nanocarriers [225, 226]. However, the direct use of VLPs is associated with many safety risks, including broad viral tropism, high immunogenicity, and pathogenicity, which significantly limits their further use [227].

**Fig. 7:**
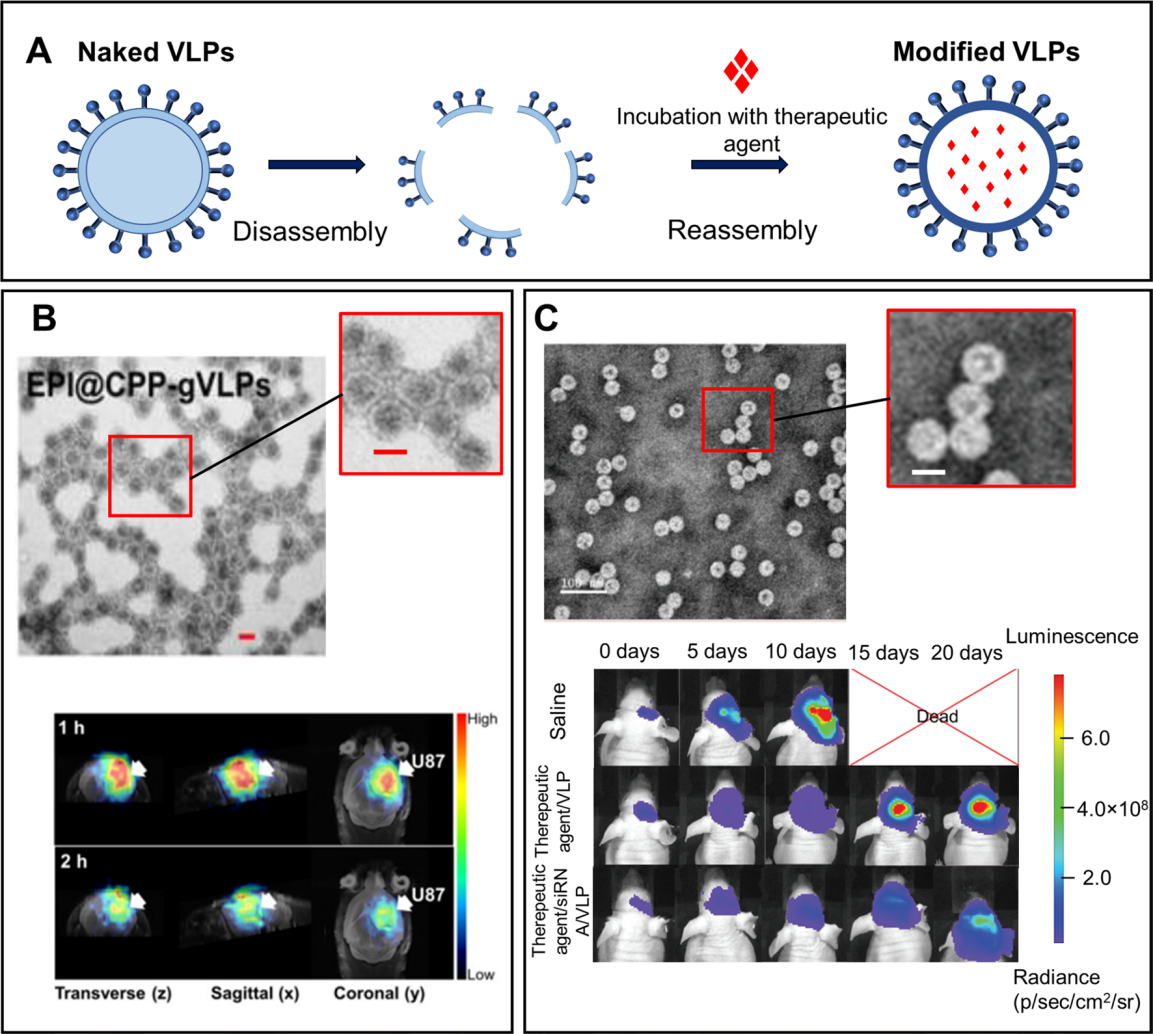
Drug loading of VLPs and their application for brain tumor delivery: **A** Schematic illustration of drug loading into VLPs. **B** TEM images of EPI@gVLPs and micro-PET-MR images of mouse brain tumors, demonstrating the effect of tumor inhibition (scale bar = 20 nm, inset: 10 nm). Adapted with permission from Ref.[228]. **C** TEM images of VLPs and bioluminescent imaging, demonstrating the effect of tumor inhibition (scale bar = 100 nm, inset: 10 nm). Adapted with permission from Ref. [233]

Similar to RV-based VLPs, VLPs based on other viruses can be developed for the delivery of therapeutic agents (Fig. 7A). An example of such VLPs was reported by Pang et al. [228]. This scientific group has developed green fluorescence VLPs (gVLPs) in E. coli to load epirubicin (EPI) to form EPI@gVLPs. These particles were additionally modified with cell-penetrating peptides and labeled with ^68^ Ga for PET-CT imaging. Labeled ^68^ Ga-EPI@gVLPs showed excellent stability in serum (size 30–40 nm); they also can degrade upon proteolytic degradation of the protein envelope, which ensures the release and clearance of the drug to minimize long-term accumulation. In vivo delivery of labeled ^68^ Ga-EPI@gVLPs demonstrated that the median survival rate was increased to more than 50 days when mice received 2 injections (once a week) compared to the control group (median survival: 26 days) (Fig. 7B).

Yang et al. studied the therapeutic effect of drug-loaded VLPs made of hepatitis B protein for the treatment of GBM [229]. Chemo- and gene-therapeutic agents paclitaxel and siRNA were loaded inside the VLPs. The obtained complexes had a size of 30–50 nm, and bioluminescent images of the glioma in vivo model clearly demonstrated an increased therapeutic effect of the paclitaxel- and siRNA-loaded VLPs (Fig. 7C). The results demonstrated the effective delivery of these therapeutic agents (paclitaxel and siRNA) to invasive tumor sites. The combination of chemo- and gene therapy revealed synergistic antitumor effects due to increased necrosis and apoptosis, and the ability to inhibit tumor invasion with minimal cytotoxicity.

For scalable production of VLPs, several parameters such as safety, reproducibility, and cost-effectiveness should be considered. The use of a bacterial expression system can be a universal option. The insect cell production system is another promising approach, which does not require a high level of safety or a special cultivation system. Plant viruses can be also used to prepare VLPs: they are non-toxic and biodegradable, can be self-assembled, and easily scaled. These properties make them an attractive alternative to other nanocarriers, such as liposomes and micelles [230]. However, it is difficult to develop a plant infection system in a conventional laboratory. Cell-free systems are also difficult to produce in laboratory conditions, but there are various kits for the expression of cell-free proteins available for sale. It can be concluded that the process of VLPs is not easy and requires special laboratory conditions, which complicates their large-scale production [231–233].

#### Outlook

The latest developments in nanotechnology and bioengineering allowed employing VLPs as carriers of therapeutic agents and vaccines [234, 235]. In spite of a limited number of studies, it was shown that VLPs are capable of delivering therapeutic agents into the brain tumor. Modern bioengineering approaches enabled the design of VLPs with various structural and functional features, which significantly expands the scope of their application. Since VLPs themselves are immunogens, they can stimulate both the innate and adaptive immune systems [213]. However, despite being promising, VLPs have certain disadvantages: for example, toxicity and immunogenicity. In addition, the high cost of VLPs should be considered, which makes their large-scale production challenging [228, 236].

### Organic NPs

Nanocarriers based on organic materials generally include polymer-based and lipid-based NPs, which can be fabricated from natural or synthetic polymers and lipids, respectively. The main advantages of polymer-based NPs are the use of biocompatible natural or synthetic polymers that are approved by FDA and can be degraded in biological microenvironments after their in vivo administration. Various polymers and lipids have been considered for drug delivery applications. In the following sections, we discuss the polymers and lipids that are generally used in the concept of brain tumor delivery, mainly focusing on GBM and glioma tumors.

#### Polymer-based NPs

##### Albumin-based NPs

**Fig. 8:**
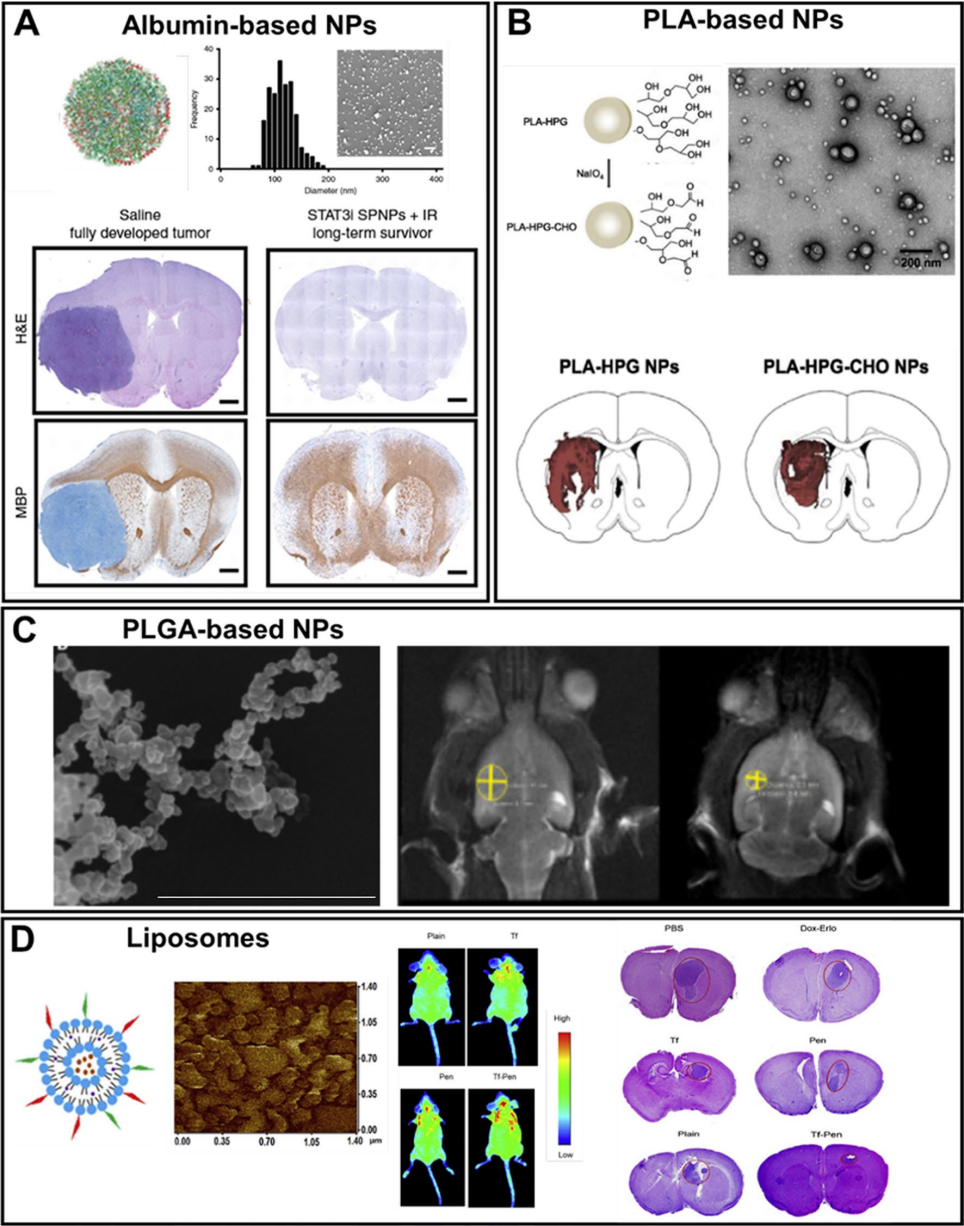
Application of organic nanocarriers for brain tumor therapy: **A** Characterization of albumin-based NPs with the following histological analysis, demonstrating tumor inhibition effect (scale bar = 1 μm). Adapted with permission from Ref. [238]. **B** Characterization of PLA-based NPs and their biodistribution in the brain (scale bar = 200 nm). Adapted with permission from Ref. [247]. **C** SEM images of PLGA-based NPs and MR images before and after the therapy (scale bar = 1 μm). Adapted with permission from Ref. [248]. **D** AFM image of liposomes, in vivo fluorescent distribution, and histological analysis, demonstrating the tumor inhibition effect. Adapted with permission from Ref. [266]

The albumin-based NPs have been extensively investigated for cancer therapy due to their unique properties, including biodegradability, non-antigenicity, and possibility of surface modification with targeting vectors. Moreover, the albumin-based NPs can also cross the BBB and reach tumor cells by SPRAC (secreted protein acidic and rich in cysteine) and gp 60 (glycoprotein 60) mechanisms-mediated targeting [237]. In the work [237], Lin et al. have found that albumin-binding proteins, i.e. SPARC and gp60, are expressed in glioma, and these pathways were applied to overcome the BBB. The authors have developed albumin-based NPs that have BBB-penetrating properties and can encapsulate different therapeutic drugs such as paclitaxel and fenretinide, exhibiting an improved treatment of glioma. Later in 2020, Gregory et al. designed protein NPs based on polymerized human serum albumin (HSA) modified with the iRGD peptide against GBM [238]. These NPs demonstrated an effective tumor delivery to GBM after their systemic administration (Fig. 8A). Recently, Kudarha et al. have prepared TMZ-loaded albumin NPs with a surface modification by hyaluronic acid (HA) to perform CD44 receptor-mediated targeting, which was used for U87 glioma treatment [239].

##### Poly(butyl cyanoacrylate) NPs

Poly(butyl cyanoacrylate) (PBCA) NPs were the first developed nanocarriers used for the delivery of biologically active compounds through the BBB [240]. Weiss et al. showed that PBCA can be used to reduce any side effects on healthy organs and demonstrate good stability both in vivo and in vitro [241]. The synthesized PBCA NPs were additionally coated with polysorbate 80 to improve their colloidal stability in biological fluids. However, it simultaneously resulted in the absorption of plasma apolipoprotein B and/or E on the surface of the developed NPs. As a result, PBCA NPs were recognized as low-density lipoproteins and possessed an enhanced uptake by the endothelial cells of the BBB via the receptor-mediated endocytosis route [242]. Later in 2014, Voigt et al. studied neuronal toxicity of PBCA NPs in vitro and in vivo [243]. According to the work, no general toxicity was found (e.g. weight loss), and no neuronal damage was detected.

Mayur and Zaved developed PBCA NPs and used them for the delivery of hydrophobic drugs such as quercetin (QT), which is known as a bioflavonoid and antioxidant with poor bioavailability and very low distribution in the brain [244]. To improve the oral bioavailability of QT and increase its distribution in the brain, a new oral delivery system consisting of PBCA NPs and the same nanocarriers coated with polysorbate-80 (P-80) was developed. The sizes of the nanoparticles were 161.1 ± 0.44 nm for the uncoated NPs and 166.6 ± 0.33 nm for the NPs coated with P-80. As a result, the relative bioavailability of QT-PBCA and QT-PBCA + P-80 NPs was increased by more than 2.38 and 4.93 times, respectively, compared with free QT. A study of biodistribution in rats showed that a higher concentration of QT was found in the brain when NPs were covered with P-80 [244].

##### Poly(lactic acid) NPs

Poly(lactic acid) (PLA) NPs have been extensively studied for drug delivery applications due to their low cytotoxicity and biodegradability. The surface of the PLA NPs can be easily modified with surfactants or targeting ligands, which overall increases the effectiveness of the BBB penetration.

In the study of Junzhu et al., the surface of paclitaxel-loaded PLA NPs was modified with cysteine–arginine–glutamic acid–lysine–alanine (CREKA) peptide that has a high affinity for fibrin to enhance tropism to GBM [245]. Due to active targeting, these PLA NPs demonstrated an improved therapeutic effect compared to free drug or unmodified nanocarriers.

In another study, the surface of PLA NPs was modified by targeting ligand – Ft peptide, which was synthesized by coupling FHK and tLyp-1 sequence together via a cysteine [246]. The synthesized Ft-modified PLA NPs demonstrated an increased affinity to ECM component tenascin C and were able to accumulate in the glioma tissue in vivo. Later, in 2019 Seo et al. designed PLA-based NPs loaded with microRNAs (miR-21) that induce cell apoptosis and prevent tumor development [247]. According to this work, the authors have employed block copolymer of PLA and hyperbranched polyglycerol (HPG) for the synthesis of PLA-HPG NPs. Additionally, the PLA-HPG NPs were further activated by NaIO_4_ to form PLA-HPG-CHO NPs. It was shown that both types of PLA-HPG and PLA-HPG-CHO NPs were distributed in large volumes in the tumor-bearing brain (Fig. 8B). These NPs loaded with therapeutic drugs demonstrated good therapeutic efficacy, prolonging the survival of animals with intracranial tumors [247].

##### Poly(lactic-co-glycolic acid) NPs

Poly(lactic-co-glycolic acid) (PLGA) is another extensively studied polymer for drug delivery. PLGA NPs are biodegradable and offer great control over the pharmacokinetics of the developed nanocarriers. Furthermore, PLGA NPs were certified by the FDA for pharmaceutical applications.

In the study of Madani et al. [248], PLGA NPs were loaded with two anticancer drugs, paclitaxel and methotrexate, to achieve a synergistic effect during cancer therapy. The surface of the synthesized NPs was modified with Poloxamer188 to promote the adsorption of apolipoprotein E and enhance the penetration across the BBB. These PLGA NPs possessed a higher antitumor activity against GBM compared with free drugs (paclitaxel and methotrexate).

In another work [249], paclitaxel-loaded PLGA NPs were additionally modified with superparamagnetic iron oxide NPs. This allowed magnetic targeting of PLGA NPs, which improved the pharmacokinetics and therapeutic effect compared to passive targeting. The developed hybrid system showed no systemic toxicity, and no signs of hepatotoxicity were detected. The use of magnetic targeting led to a significantly prolonged (49 days) survival time of tumor-bearing mice compared to control mice (41 days). Recently, Caban-Toktas et al. have designed PLGA NPs loaded with R-flurbiprofen and paclitaxel for combination therapy against glioma, using the rat RG2 glioma tumor model [250]. The MR images clearly demonstrated the tumor inhibition for PLGA NPs after the post-treatment procedure (Fig. 8C).

#### Lipid-based NPs

Recently, lipid-based NPs have gained significant interest due to their biodegradability, non-toxicity, excellent ability to target tumors, and surface modification possibilities, as well as the highly efficient encapsulation of lipophilic drugs. Lipid NPs include lipid nanocapsules, nanosomes, liposomes, micelles, and solid lipid NPs. All the listed carriers consist of lipids and include an oil solution, suspension or emulsion [251].

Liposomes are a classic example of lipid-based NPs. Liposomes are spherical vesicles that consist of one or more concentric bilayers of phospholipids surrounding an aqueous phase. Being non-toxic and biodegradable, liposomes are a powerful drug delivery system. Liposomes mainly consist of glycerophospholipids, which are amphiphilic lipids composed of a glycerol molecule attached to a phosphate group and two chains of either saturated or unsaturated fatty acids [252–255].

Due to the amphiphilic properties of phospholipids, they tend to self-assemble and form stable bilayer structures in aqueous environment. This process is facilitated through the hydrophilic interactions between polar head groups, Van der Waals forces between nonpolar hydrocarbon chains, and hydrogen bonds with surrounding water molecules. Hydrophobic chains are repelled by polar water molecules, and liposomes spontaneously self-organize into an enclosed bilayer [256].

Liposomes can encapsulate both hydrophilic and hydrophobic drugs. For this reason, liposomes are extensively studied as drug delivery systems. Despite their hydrophobic nature, liposomes cannot simply diffuse through the BBB due to their size. They can cross the BBB via AME, RME, and CMT. To efficiently utilize the above-mentioned mechanisms for drug delivery across the BBB, further surface modification of liposomes is required. For this, the surface of liposomes is complemented with cations, PEG, antibodies or other ligands.To make liposomal drug delivery site-specific, the surface of such particles is usually modified with targeting ligands. The most commonly used approach for such a modification utilizes various antibodies that target the antigens expressed at the surface of glioma cells [257, 258]. For example, anti-TfR single-chain antibodies have been used to decorate liposomes and specifically target GBM through the BBB [259, 260]. Another approach to increase the specificity of liposomes is to modify their surface with proteins (such as transferrin [261, 262]) or peptides (such as cell-penetrating peptides [263, 264]).

In 2009, Beier et al. demonstrated the efficiency of combination therapy based on PEGylated liposomal DOX, TMZ, and radiation therapy. As a result, PEGylated DOX loaded in the liposomal membrane was shown to penetrate the BBB more effectively compared to free drugs, and the median survival of animals increased up to 17.6 months [265]. Then, Lakkadwala et al. continued working in the field of combination therapy against brain tumors. In particular, a detailed investigation was performed of the use of drug co-loaded (DOX and erlotinib) liposomes modified with transferrin (Tf) for receptor-mediated endocytosis and a cell-penetrating peptide, penetratin (Pen), against GBM, using tumor mice model [266]. The in vivo imaging of mice clearly showed the accumulation of fluorescent-labeled liposomes in the brain (Fig. 8D). The excellent targeting of liposomes and their penetrating ability into GBM led to a significant decrease in tumor growth (Fig. 8D).

Previous studies have demonstrated that low-intensity focused ultrasound (LIFU) combined with systemic injection of lipid-shelled microbubbles can induce a noninvasive, local, and transient disruption of the BBB [267, 268]. Based on this, Lin et al. employed DOX-loaded cationic liposomes combined with LIFU for the BBB penetration and targeting C6 glioma in a rat model [269]. The use of LIFU induced BBB opening so that liposomes could deliver DOX into glioma. This combined procedure led to prolonged glioma inhibition with minimal side effects. Later in 2019, Papachristodoulou et al. reported that LIFU can mediate effective delivery of liposomes to the tumor region, which was demonstrated on mice bearing TMZ-resistant gliomas [270]. Very recently, Morse et al. applied a rapid short-pulse (RaSP) ultrasound for the delivery of drug-loaded liposomes to the murine brain in vivo [271].

#### Outlook

Organic NPs can be also applied as carriers of therapeutics to treat brain tumors, in particular, GBM. The main advantage of organic NPs is their high biocompatibility and biodegradability, which can regulate the release of therapeutic agents [272, 273]. Simple synthetic routes with the possibility of varying the size, shape, and functionality of organic NPs significantly increase their potential in the treatment of different types of brain tumors. However, organic NPs have some disadvantages, including low stability in biological fluids and sensitivity to storage conditions [272]. Furthermore, when organic NPs are introduced into the systemic circulation, blood plasma proteins are adsorbed on them. It can lead to enhanced clearance of NPs from the blood through the absorption-excretory system of the spleen and liver, which prevents their accumulation in the required tumor area, reducing the targeting ability [274]. The problems of organic NPs’ stability in liquid media and their protein adsorption can be solved by using surfactants (tween, poly(ethylene–glycol), D-a-tocopheryl polyethylene glycol) [275].

### Inorganic NPs

Inorganic NPs, such as silica (SiO_2_), gold (Au), iron oxide (IONPs), silver (Ag), and others, are extensively studied as drug delivery carriers due to their well-controlled physicochemical properties, which can be tuned during synthesis. Furthermore, inorganic NPs can be considered not only as carriers of drugs but also as an independent unit for imaging methods (MRI, CT) [276].

#### SiO_2_ NPs

**Fig. 9:**
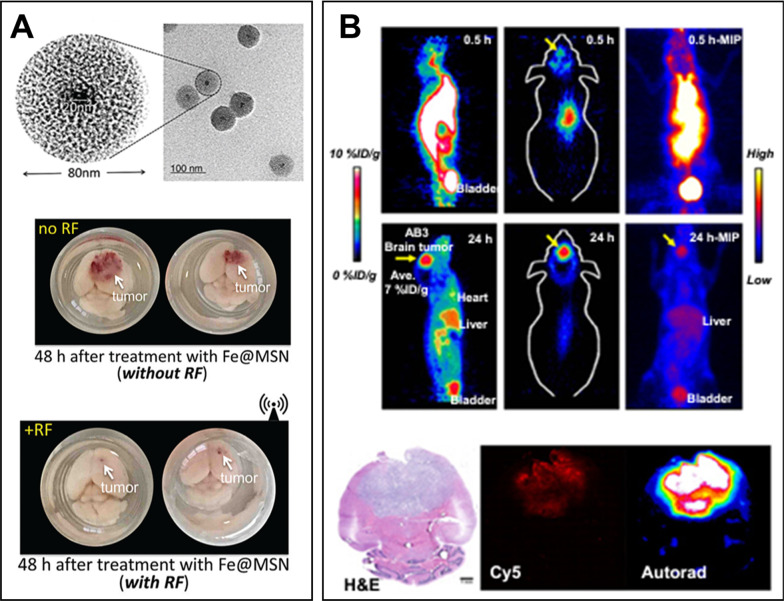
Application of SiO_2_-based NPs for targeting brain tumor: **A** TEM images of IONPs@SiO_2_ NPs with corresponding macroscopic ex vivo evaluation of their therapeutic efficiency against GBM tumors (scale bar = 100 nm). Adapted with permission from Ref. [279], **B** PET-CT imaging of radiolabeled core–shell SiO_2_ NPs showing clear accumulation of radionuclide signal in the brain tumors with corresponding histological and fluorescent analysis of NPs accumulation in the brain. Adapted with permission from Ref. [280]

Drug delivery systems based on SiO_2_ NPs can be considered as another alternative to deliver bioactive compounds to brain tumors. Due to their porosity, SiO_2_ NPs have a large surface area, which can be used for increased drug loading. Furthermore, the size, shape and pore sizes of SiO_2_ NPs can be tuned by changing the synthesis conditions [277]. Colloidal SiO_2_ is utilized in medical tablet manufacturing and is recognized as safe by the FDA. At present, various SiO_2_ NPs formulations are being tested in phase I and II clinical trials [278]. In general, SiO_2_ NPs are often combined with other components to obtain multicomponent drug formulations. They can be easily modified with IONPs and Au NPs. For example, Turan and co-workers developed multicomponent SiO_2_ NPs consisting of an iron oxide core and mesoporous silica shell that can effectively deliver drugs across the BBB into glioma cells [279]. The surface of the multicomponent SiO_2_ NPs was coated with fibronectin to provide active targeting to glioma cells. The drug release from the NPs was achieved by external radiofrequency (RF) fields; this therapy resulted in a twofold increase in animal survival (Fig. 9A). Next, Juthani et al. prepared ultrasmall core–shell SiO_2_ NPs for GBM treatment [280]. By employing fluorescent dyes and diagnostic radionuclides, the nanocarriers were proven to penetrate brain tumors with high efficiency (Fig. 9B).

Besides inorganic components, the SiO_2_ NPs can be modified with a lipid layer. For instance, Zhu and colleagues reported on the use of angiopep-2-modified lipid-coated SiO_2_ NPs for glioma targeting therapy overcoming the BBB [281]. This lipid-coating has led to an improved targeting efficiency of paclitaxel (20.6%) compared to the non-modified NPs (10.74%) that provided a prolonged survival time of C6 glioma-bearing rats (from 20 to 30 days). Another research group modified SiO_2_ NPs with PLGA. This modification allowed the encapsulation of paclitaxel as an anticancer drug and greatly enhanced the anticancer efficacy [282].

#### Au NPs

Au NPs are metallic colloidal NPs that have found numerous applications in drug delivery. They demonstrate great biocompatibility, and both their size and shape can be easily modified to alter their biodistribution [283, 284]. Among noble metals, Au NPs have found important applications in photothermal therapy (PTT) because they support localized surface plasmon resonances (LSPR). The LSPR effect can facilitate the absorption of light by Au NPs and convert the absorbed light into heat. However, during PTT, the energy of the absorbed light is only partially converted into heat for the elimination of cancer cells in vivo [285]. Apart from applying Au NPs as nanoheaters, they can be used as contrast agents in X-ray imaging due to their high X-ray attenuation [286]. The highly reactive surface of Au NPs can be modified with a variety of targeting ligands or biologically active compounds. The size of Au NPs can be greatly altered from 1 to > 200 nm to suit the needs of drug delivery. Depending on the size and surface modification of Au NPs they can be transported across the BBB through passive diffusion, RME, AME, and CMT [287, 288].

**Fig. 10:**
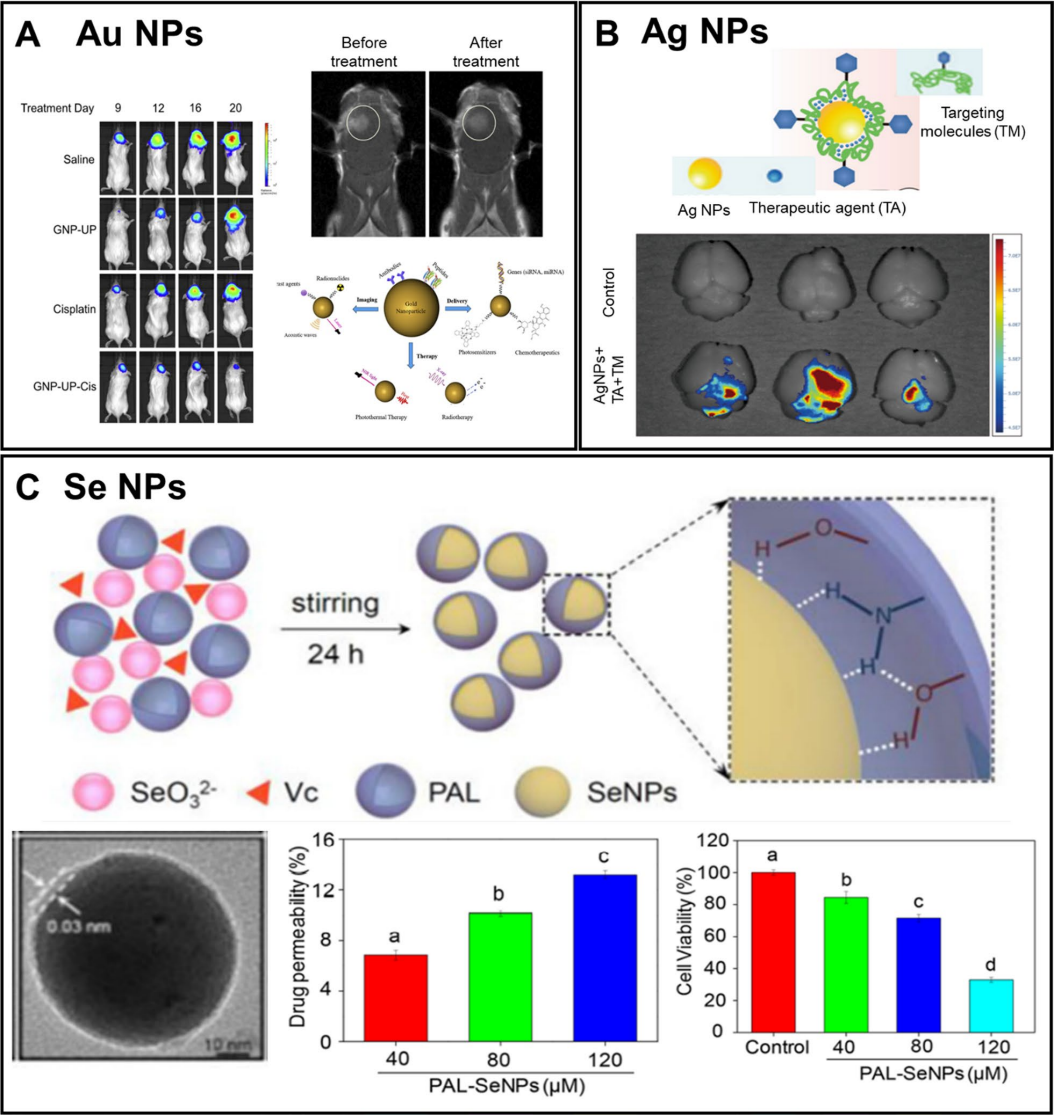
Application of inorganic nanocarriers based on Au, Ag, and Se NPs for targeting of brain tumor: (**A**) Bioluminescent images of the tumor showing the specific accumulation of Au NPs in the glioma and the anti-tumor effect after therapy. Adapted with permission from ref. [289], **B** Schematic illustration of the design of Ag NPs and ex vivo imaging of the accumulation of Ag NPs in the brain tumor. Adapted with permission from Ref. [310], **C** Schematic illustration of the synthesis of glioma cell targeting complexes based on Se NPs, TEM image of Se NPs and histograms of drug permeability and cytotoxicity (scale bar = 10 nm). Adapted with permission from Ref. [298]

Au NPs have demonstrated their efficiency in the delivery of antitumor drugs. A 2018 study by Collucia et al. (Fig. 10A) described the use of Au NPs conjugated with cisplatin in combination with MR-guided focused ultrasound for successful in vivo inhibition of GBM growth [289].

Additionally, Au NPs can be utilized to enhance the efficiency of radiotherapy. In 2021, Dong and colleagues designed a radiotherapy sensitizer based on sub-nanometer Au NPs, BBB-penetrating peptide iRGD, and cell cycle regulator α-difluoromethylornithine [290]. Due to the high atomic number of Au, it can absorb X-ray efficiently and boost radiotherapy. The use of the developed Au NPs allowed low-dose radiotherapy to achieve the same efficiency of treatment as high-dose radiotherapy, with a significantly reduced fatality rate, which is a promising approach for the treatment of GBM.

#### Se NPs

Currently, Se NPs have drawn significant attention as therapeutic agents [291]. There are reports describing the antitumor activity of Se NPs due to their effect on reactive oxygen species (ROS) generated inside cells [292–296]. Internalization of Se NPs by tumor cells increases the production of mitochondrial ROS that results in ATP depletion and consequent cell death. Furthermore, Se NPs activate tumor necrotic factor and interferon regulatory factor that induces necroptosis through receptor-interacting protein 1 [297].

Chen et al. developed *Polyporus Amboinensis Lam* (PAL)-functionalized Se NPs to pass through the BBB and thus enhance the therapy of glioma (Fig. 10C) [298]. PAL-Se NPs up-regulated the ROS level and induced apoptosis in U87 glioma cells, which was demonstrated by in vitro and in vivo analysis. Similarly, Song et al. synthesized “smart” nanosystems based on Se NPs modified with HER-2 antibody for effective delivery into the brain tumor [299]. Wenjian has developed inorganic hybrids made of Ag and Se NPs (Ag@Se NPs) for the GBM therapy [300]. Ag@Se NPs were conjugated with RGD peptides (Ag@Se-RGD NPs) for GBM targeting. The results showed that Ag@Se-RGD NPs possessed high in vitro stability and induced a significant intracellular uptake by human glioma cells U251 with the following cell apoptosis due to ROS effect [300].

Despite the promising results of Se NPs for therapy of brain tumor, currently, most of the existing studies were conducted in vitro. The data demonstrating their effectiveness for in vivo delivery through the BBB is rather limited, and further research is required.

#### Ag NPs

Ag NPs are another type of nanocarriers for drug delivery application, which have unique optical properties. Similar to Au NPs, Ag NPs possess LSPR, which can be applied to induce apoptosis in cancer cells via heating [301–303]. The absorption efficiency of light by Ag NPs mainly depends on the geometry of NPs [285, 304].

Ag NPs can inhibit tumor growth and induce apoptosis of cancer cells [305–307]. The mechanism of tumor inhibition has not yet been fully investigated. Ag NPs can induce DNA damage, preventing DNA synthesis and further leading to cell cycle blocking in G2/M phase and apoptosis of tumor cells [308]. Furthermore, Ag^+^ ions can disrupt calcium homeostasis, which results in an increased toxicity of Ag NPs [309].

Jianming et al. investigated Ag NPs loaded with albendazole (Abz). For targeted therapy of glioma, Abz-loaded Ag NPs were coated with BSA. According to Fig. 10B, the obtained Ag-based hybrids have the ability to overcome the BBB and accumulate in the glioma [310]. In this study, modification of Ag NPs with Abz significantly increased the median survival time of glioma-bearing mice (from 15 to 23 days) with no toxicity on healthy tissues.

In contrast, various works demonstrate that Ag NPs can damage healthy tissues and induce systemic toxicity. For example, Ag NPs can increase the permeability of the BBB and suppress the antioxidant protection of astrocytes by increasing the protein interaction with thioredoxin, which leads to central neurotoxicity [311, 312].

#### IONPs

IONPs are of considerable interest as drug carriers due to their colloidal stability and low toxicity. IONPs have size-dependent magnetic properties, which are used for their navigation under an external magnetic field [313]. An interesting feature of iron IONPs is their ability to heat under an alternating magnetic field. This property of IONPs is actively used in the so-called magnetic hyperthermia. Notably, the heating rate of IONPs depends on size, shape, monodispersity and quality of NPs [314]. IONPs can be coated with biocompatible and biodegradable polymers to form a core–shell structure [315, 316]. To pass the BBB, the particle size can be adjusted from 5 to 100 nm, depending on the synthetic conditions, and the surface of IONPs can also be modified with targeting molecules. Several studies have demonstrated that IONPs can cross the BBB by applying an external magnetic field [317, 318]. For instance, Qiu et al. developed IONPs modified with phospholipid-PEG and showed the use of magnetic forces for controlled drug delivery by disrupting endothelial cell–cell junctions [319]. This finding provides some valuable technical data relevant for overcoming the BBB using an external magnetic field. In another study, Huang et al. demonstrated IONPs coated with PEG, PEI, and Tween-80 efficiently crossing the BBB in the rats by applying an external 0.3 T magnetic field [320].

**Fig. 11:**
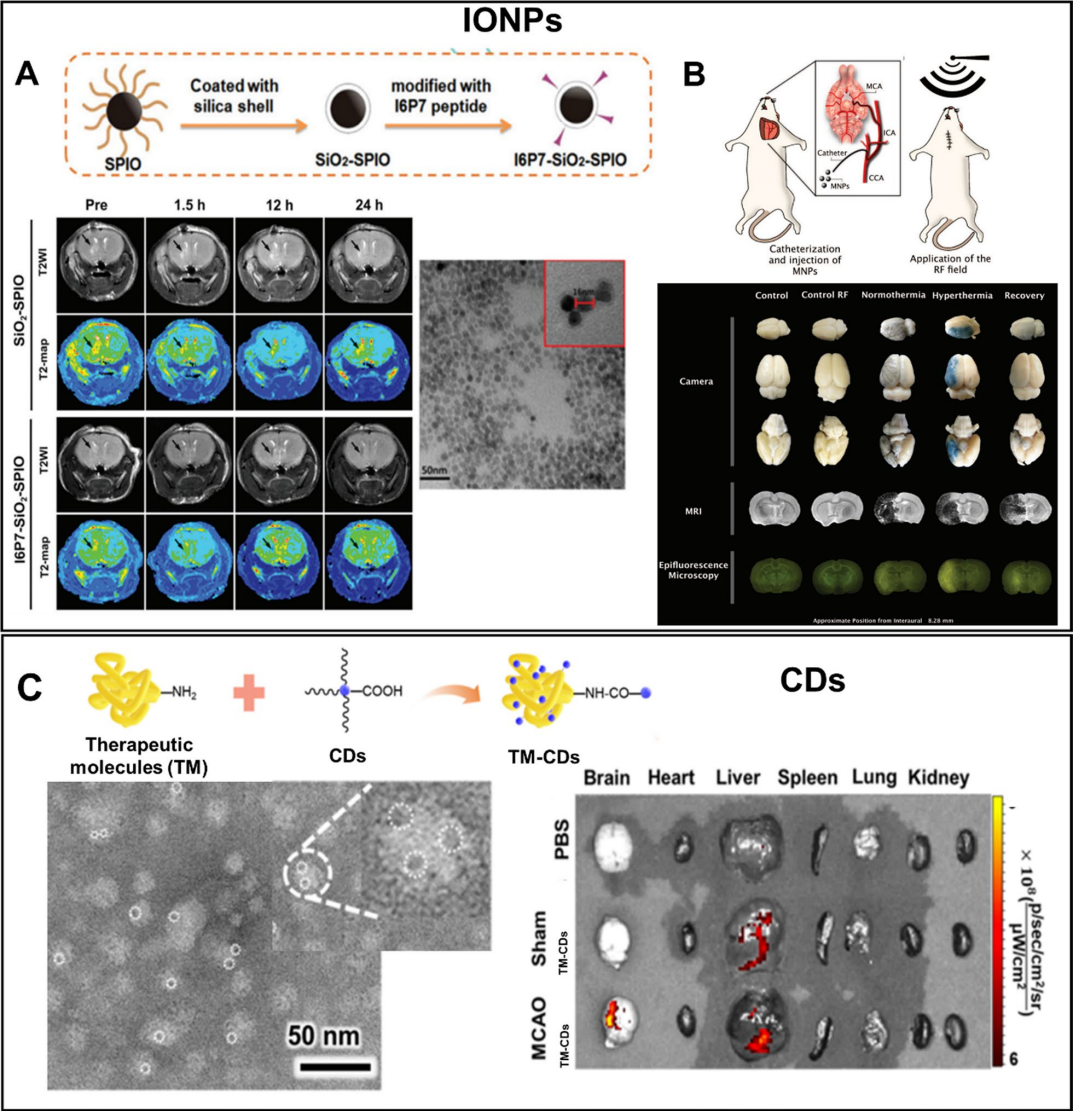
Application of inorganic nanocarriers based on IONPs and CDs for targeting brain tumor: **A** Schematic illustration of the design of the hybrids based on IONPs and corresponding TEM, MR, and PET-CT images demonstrating the delivery efficacy of IONPs to the brain (scale bar = 50 nm, inset: scale bar = 16 nm). Adapted with permission from Ref. [323], **B** Schematic illustration of overcoming the BBB using an adjustable RF field to perform hyperthermia with commercially available IONPs (injected into the external carotid artery). MR and fluorescent images showing the delivery of IONPs to the mouse brain. Adapted with permission from Ref. [324], **C** Scheme of synthesis and TEM image of hybrids based on CDs. Ex vivo analysis of the main organs (the brain, heart, liver, spleen, lungs, and kidneys) of mice after a 30-min intravenous injection, demonstrating the accumulation of nanohybrids in the brain (scale bar = 50 nm). Adapted with permission from Ref.[333]

Considering active targeting mechanisms, there are plenty of works describing the modification of IONPs with targeted molecules to facilitate their transport to the brain tumor [321]. For instance, Chen et al. synthesized IONPs conjugated with BBB-penetrating peptide anigiopep-2 (ANG) [322, 323]. Recently, Tan et al. developed novel IONPs covalently modified with interleukin-6 receptor-targeting peptides (I6P7) to provide delivery through the BBB and recognition of gliomas (Fig. 11A) [323]. Further, in vivo studies demonstrated an efficient accumulation of I6P7-modified IONPs in the tumor region, proved by MR imaging.

Several methods of IONPs delivery through the BBB were implemented without active targeting, using non-specific mechanisms. One of these approaches is to locally open the BBB for a certain time, using an adjustable RF field to achieve hyperthermia with commercially available IONPs injected through the medial cerebral artery (Fig. 11B) [324]. Lammers et al. described another method for the delivery of IONPs across the BBB [325]. In particular, the authors used poly(butyl cyanoacrylate)-based micro-bubbles loaded with IONPs that can mediate the BBB penetration. When exposed to ultrasound, the micro-bubbles were destroyed, which led to the release of IONPs; this caused acoustic forces, increasing the vascular permeability of the BBB. As a result, the IONPs released from microbubbles could penetrate the permeabilized BBB and accumulate in extravascular brain tissue.

Wu et al. developed an aqueous ferrofluid without surfactants, containing superparamagnetic IONPs (SPIONs) coated with SiO_2_ and carbon shells with an average size of 13 nm [326]. The double coating of SPIONs significantly increased their colloidal stability in biological fluids. The developed SPIONs reduced the viability of GBM and osteosarcoma cells. They showed an enhanced targeting of cancer cells due to the increased absorption abilities of these NPs and their pronounced adhesion to the membrane of cancer cells. The death of tumor cells occurred due to the heating of IONPs exposed to a magnetic field. Even in an ultra-low alternating magnetic field, the NPs released enough heat to cause tumor death [326].

#### CDs

Carbon dots (CDs) are a broad class of carbon-based nanomaterials that include carbon nanodots, graphene quantum dots (GQDs), and carbon nitride dots (CNDs) [327]. Several techniques of CDs synthesis have been developed, mainly focusing on top-down and bottom-up approaches [328]. The unique optical properties of CDs open a wide potential for diagnostic purposes. Indeed, CDs can demonstrate excitation-dependent emission from the blue-green range of light to the orange-red and near-infrared ranges [329, 330]. They also possess a high fluorescence quantum yield. Several techniques were developed to modify the surface of CDs with functional groups, such as − COOH, − OH, − C = O, and − C = N [331]. The functional groups on the surface of CDs allow their further modification with targeting vectors or biocompatible polymers for the design of targeted drug delivery platforms. A significant interest in the application of CDs for crossing the BBB has started after 2015, and now these researches are rapidly growing [327]. To examine the ability of CDs and their hybrids to overcome the BBB, in vitro and in vivo models have been developed. At present, the mechanisms explaining how CDs cross the BBB remain poorly understood. Despite this fact, three main mechanisms of CDs penetration across the BBB can be distinguished. The first one includes passive diffusion. The size of CDs can be less than 4 nm, which allows passing the gap between the endothelial cells of the BBB (4–6 nm). For instance, Lu et al. have prepared CDs with a size of around 2.6 nm and with a high quantum yield (51%) [332]. To study the BBB-penetration ability of these CDs, a biomimetic BBB model created from rat brain microvascular endothelial cells (rBMEC) and astrocytes was employed. It was found that the CDs were able to cross the BBB in a concentration-dependent manner [332]. Yuefang Niu et al. demonstrated the accumulation of CDs in the brain of mice using fluorescent imaging analysis of ex vivo organs (Fig. 11C) [333].

Another way to overcome the BBB is the use of electric charges. Thus, CDs with the size of 2.6 nm were additionally coated with cationic polyethyleneimine (PEI), enhancing the penetration ability through the BBB [332–334]. The third approach is based on the RME mechanism, which is the most classic strategy for overcoming the BBB. For instance, Hettiarachchi et al. developed triple conjugated CDs (the size was 1.5–1.7 nm) modified with transferrin (the targeted ligand) and two anti-tumor drugs (epirubicin and temozolomide) [335]. The transferrin-conjugated CDs showed a higher affinity to U87 glioma cells compared to non-modified CDs. In another study by Qiao et al., the authors developed the CDs modified with d-glucose and l-aspartic for targeting brain tumor cells (C6 cells) [336]. Recently, Li et al. prepared exosome-coated ^10^B-CDs for BNCT of brain tumors, which was demonstrated on a glioma mice model [337]. The excellent accumulation of these CDs in the brain glioma in vivo allowed an effective BNCT, achieving a prolonged survival rate of mice compared to control samples (a survival ratio of 100% for 30 days in case of BNCT and a survival ratio of 0% after 15 days of experiments in case of control group, i.e. mice without therapy) [337].

#### Outlook

Inorganic NPs have unique physico-chemical properties, such as nanometric size and increased loading capacity (e.g., SiO_2_ NPs). Inorganic NPs, for example, gold, iron-oxide, and carbon-based ones, have magnetic, electrical, optical and thermal properties that allow them to be used as contrast agents [338, 339]. Therefore, inorganic NPs can be applied in multimodal imaging, as well as theranostics [340–344]. These NPs can also actively absorb NIR energy and generate heat at the same time, which further improves their therapeutic effect and increases the optical resolution for analytical instruments [345]. Compared to the commercially available contrast agents (e.g. magnevist, omniscan), inorganic NPs possess a longer systemic circulation within the body [346, 347]. Due to LSPR, Au NPs are well-suited for optical coherence tomography [348] and computer tomography imaging [349]. IONPs can be used for MRI imaging [350].

### Cell-based delivery systems

Recently, cell-based delivery systems have received increased interest due to their potential for tumor applications. The main feature of such systems is their ability to mimic specific properties of organisms and deceive the system, providing site-specific delivery of therapeutic drugs [351]. In this context, the use of cells as carriers is a promising strategy for overcoming the BBB for the delivery of therapeutics to the brain tumor [352]. Various cells with brain tumor tropism have been investigated as carriers, including mesenchymal stem cells (MSCs), macrophages, neutrophils, erythrocytes, leukocytes, and monocytes [353]. However, drug loading into these cells is a challenging task. At present, four pathways for drug loading into cells can be considered: (i) non-covalent binding, (ii) covalent binding to cell membrane, (iii) internalization via cell endocytosis, and (iv) specific antibody-antigen interactions [211].

Besides the use of individual cells, another strategy employs cell components, i.e. cell membrane, for surface coating of nanocarriers. The interactions between a specific cell and brain endothelial cells can be used for developing cell membrane-coated nanocarriers that can cross the BBB and reach brain tumors. Extracellular vehicles (EVs) are an additional type of cell-based delivery system [351]. The capacity of EVs to overcome natural barriers, such as the BBB, was effectively used for delivery into brain tumors [351]. However, the encapsulation of hydrophilic/hydrophobic drugs into EVs can be difficult, and it can be realized by other, simpler strategies [351]. In the following sub-chapters, various cell-based delivery systems for targeting brain tumors will be presented and discussed.

#### MSCs-based vehicles

It is well-known that MSCs can migrate to the tumor site due to their tropism associated with the stromal cell-derived factor-1 (SDF-1)/CXC chemokine receptor type-4 (CXCR4) axis [354]. The possible mechanism of the cell homing effect is the following: the high level of SDF-1 secreted by tumor cells serve as a chemoattractant gradient for MSCs, expressing CXCR4 receptor [355]. In many studies, to improve the homing effect of MSCs, they are genetically modified with overexpression of CXCR4 receptors [356]. For this purpose, the lentivirus is usually used. For example, MSCs transfected by lentiviral vectors (LV) carrying the gene of the fourth chemokine receptor (CXCR4) contributed to the migration to damaged areas due to the overexpression of CXCR4 [357, 358]. Genetically modified MSCs expressing an apoptosis-inducing ligand associated with tumor necrosis factor (TNF) (TRAIL) demonstrated targeted delivery and local production of TRAIL at the glioma tumor site [358, 359].

**Fig. 12:**
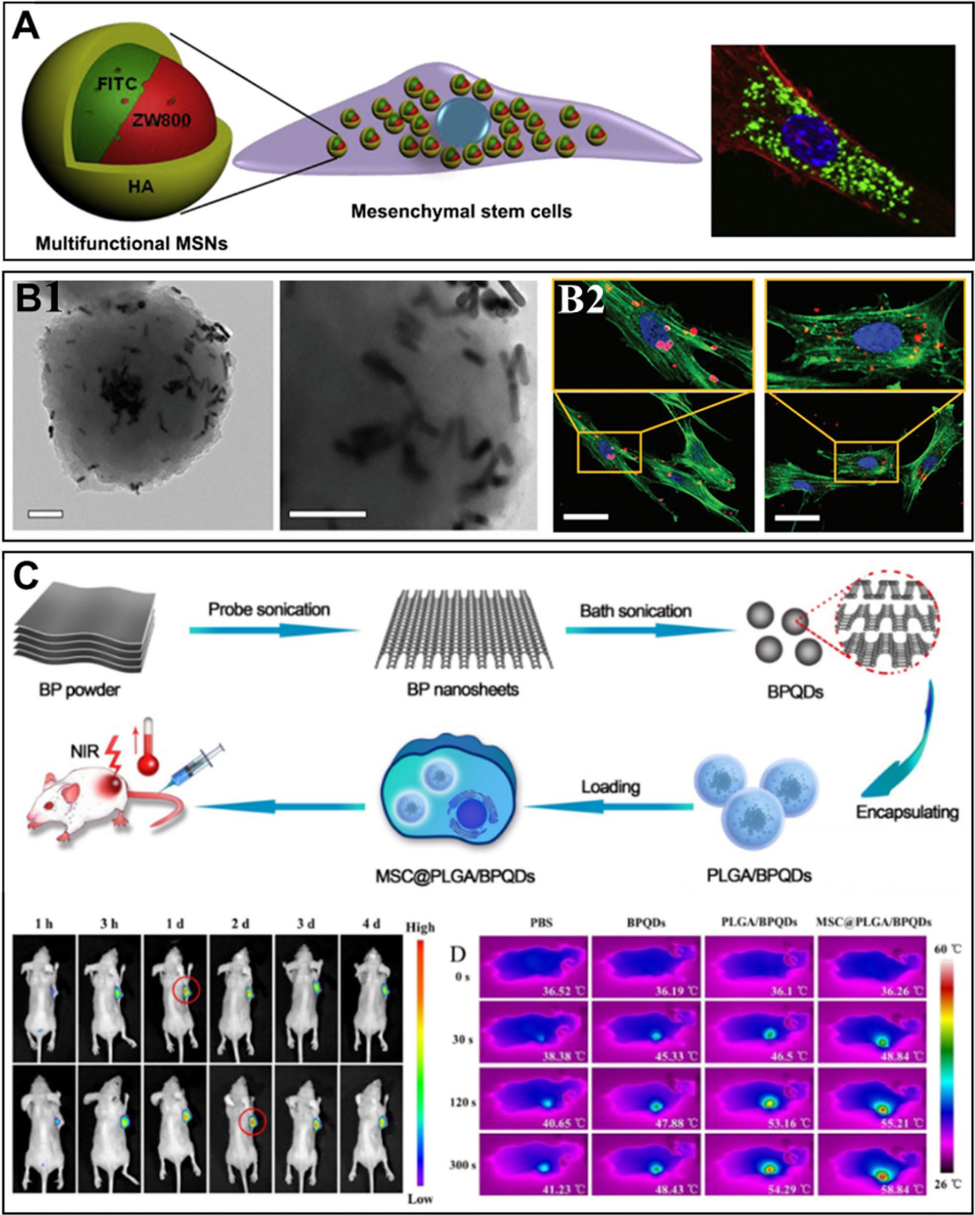
MSCs-based vehicles for overcoming the BBB and delivery to the glioma: **A** Schematic illustration and characterization of MSC-based multifunctional cell platform (MSC-platform). Adapted with permission from Ref. [359], **B** MSCs-delivery system modified with NIR-responsive drug carriers (B1: scale bar = 100 nm, B2: scale bar = 20 μm). Adapted with permission from Ref. [361], **C** Schematic design of PLGA/BPQD-loaded MSCs for enhanced photothermal therapy of U251 glioma. Adapted with permission from Ref. [362]

One of the most cited works about MSCs for the delivery of NPs to brain tumors was reported by Roger and co-workers (Fig. 12A) [359]. The authors demonstrated the proof-of-concept that MSCs can be used for the delivery of NPs loaded with therapeutic drugs into brain tumors. Another study by Huang et al. reported on MSCs modified with Si NPs as a multifunctional platform for targeted delivery to the brain tumor (U87MG mice model) [359]. They used the non-covalent binding of SiO_2_ NPs to the cell membrane. A similar approach of MSCs modification was used in the more recent works (Fig. 12B) [360, 361] By combining nanocarriers with different properties for MSCs modification, several therapeutic methods of brain tumor delivery can be realized. For instance, Luo et al. incorporated PLGA/black phosphorus quantum dots (BPQDs) in the MSCs for effective targeted photothermal therapy of U251 glioma tumors (Fig. 12C) [362].

#### Erythrocyte-based vehicles

Erythrocytes, or red blood cells (RBCs), are the major components of blood and the main carriers of oxygen to the tissues via blood flow through the circulatory system. They possess several properties making them suitable for drug delivery [353]. However, the use of individual cells for drug delivery to mediate cancer treatment is not sufficient, because erythrocytes cannot migrate across the endothelial barrier and therefore deliver the drug to the tumor zone [363]. For tumor brain targeting, NPs are coated with cell membranes from erythrocytes to provide their long blood circulation with low immunogenicity [363]. For active targeting to brain tumors, the surface of cell-based nanocarriers should be additionally coated with targeted molecules, i.e. peptides. For example, Fu et al. designed RBC membrane-coated solid lipid NPs modified with T7 and NGR peptides [364]. The vincristine was used as a model antitumor drug for loading into the NPs. After encapsulation of vincristine, the cell membrane-coated carriers exhibited the most favorable anti-glioma effects in vitro and in vivo, increasing the survival rate of laboratory animals. In another work, Chai et al. reported on an effective delivery system consisting of robust RBC membrane-coated NPs modified with CDX peptide (RBCNPs-CDX) [365]. The authors demonstrated that the DOX-loaded RBCNPs-CDX possessed a more significant therapeutic efficacy and reduced toxicity compared to the non-targeted DOX for a glioma mouse model. Further, Cui reported on the preparation of erythrocyte membrane-coated PLGA NPs dual-modified with WSW and NGR peptides [366]. In vivo studies on glioma mouse models have shown that after intravenous injection, these NPs could enter the brain, target the tumor tissue, and significantly increase the survival rate. In another recent report by Chai et al., a targeted delivery system made of drug nanocrystals and modified with RBC membrane targeting tumor-peptides is described [367].

#### Immune cell-based vehicles

Nowadays, the use of immune cells, i.e. neutrophils, lymphocytes, and mononuclear phagocytes such as dendritic cells, monocytes, and macrophages, is one of the most promising approaches for cancer treatment. It was demonstrated that immune cells can cross the BBB [368]. Therefore, these cells and their components have been explored for the delivery of therapeutic drugs into brain tumors. Moreover, immune cells are used in the immunotherapy of malignant brain tumors [368, 369] Brain tumors are characterized by an inflammatory process and extensive recruitment of immune cells, mainly macrophages and T-cells, which can immediately respond to inflammation and migrate across the BBB into pathogenic tissues [211]. For example, Pohl-Guimaraes et al. showed that RNA-modified T cells can deliver immunomodulatory agents directly to brain tumors. The obtained results clearly demonstrated a significantly extended overall survival in an orthotopic treatment model, proving effective delivery of biological agents across the BBB using T-cells. However, for most patients, endogenous immune responses are not strong enough to create sufficient anti‐tumor responses, and thus, the engineering of new T-cell immunity using chimeric antigen receptors (CARs) is a rapidly developing approach for the treatment of cancer [370]. Early investigations using CAR T therapy against brain tumors have shown great promise, even though there are some challenges, including addressing tumor antigen heterogeneity, overcoming an immune‐suppressive tumor microenvironment, ensuring sufficient T-cell trafficking to the tumor, enhancing CAR T-persistence, and avoiding toxicity [370]. In this regard, Brown et al. developed a CAR T-cell immunotherapy targeting IL-13 receptor α2 (IL13Rα2) for the treatment of GBM [371]. Recently, Donovan et al. evaluated locoregional CSF delivery of CAR T-cell therapy as a treatment approach in xenograft mouse models of metastatic medulloblastoma [372].

The tumor-homing mechanism of macrophages and monocytes also makes these cell types promising candidates for brain tumor therapy. An additional advantage of macrophages is their phagocytic capabilities that allow the internalization of different types of NPs loaded with therapeutic compounds. Pang et al. demonstrated the feasibility of macrophages loaded with Dox-NPs to deliver drugs into glioma [373, 374]Recently, Ibarra et al. assessed a delivery system based on monocytes conjugated with polymer NPs for enhanced photodynamic therapy of GBM [375], which showed a promising targeting efficiency to GBM.

**Fig. 13:**
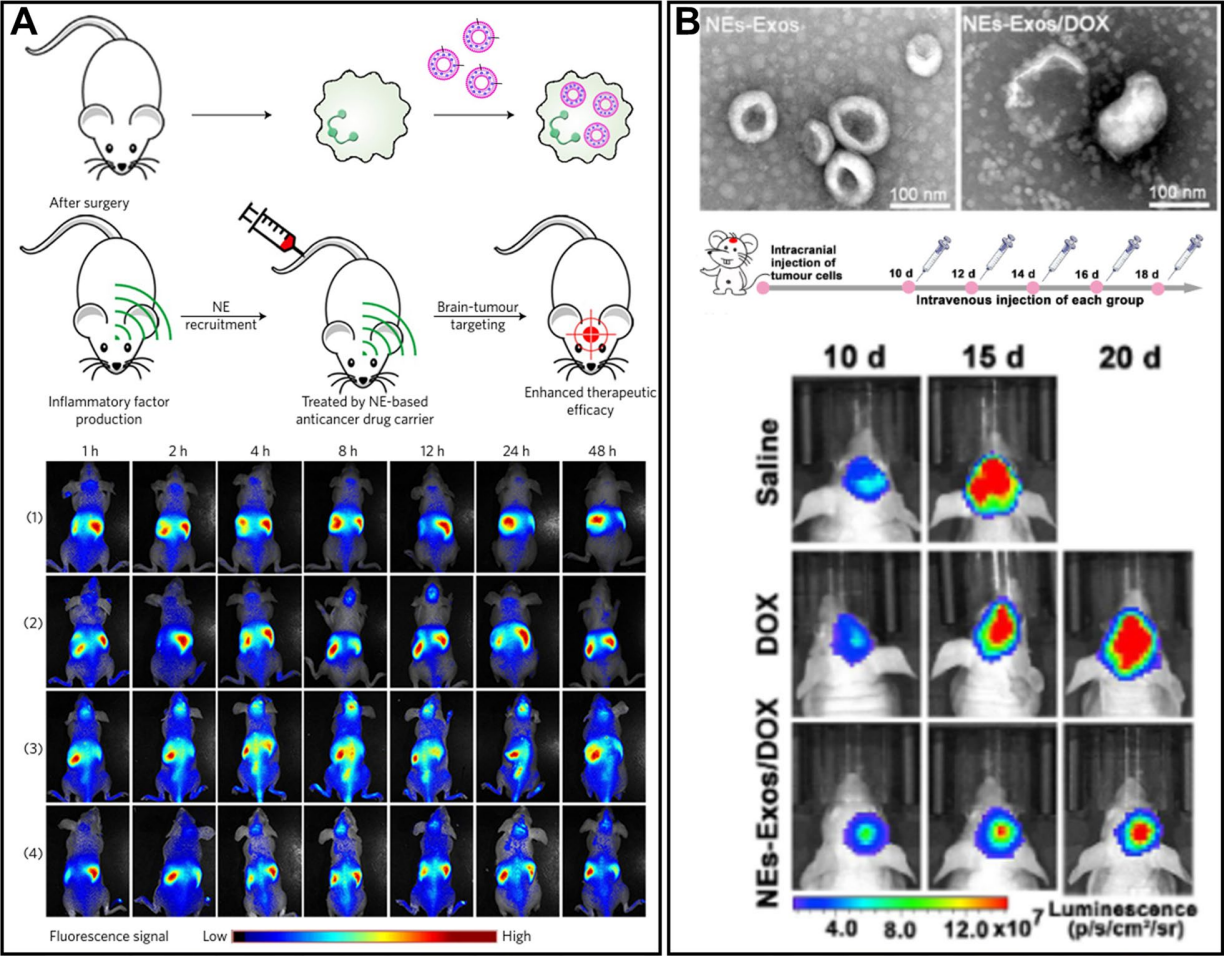
Neutrophil-based delivery systems for overcoming the BBB and penetration into the brain tumor: **A** Schematic illustration of the preparation of neutrophils modified with paclitaxel-loaded liposomes to target glioma tumors in mice. Below, in vivo fluorescence imaging of the normal and glioma-bearing mice after intravenous administration of neutrophils-based vehicles is shown. Adapted with permission from Ref. [377]. **B** TEM images of the neutrophil-exosomes system for the delivery of the antitumor drug DOX into the glioma (scale bar = 100 nm). Below, in vivo results (6-Luc glioma-bearing mice model) demonstrate that this cell-based vehicle can transport the drug through the BBB and migrate into the brain tumor with high efficiency. Adapted with permission from Ref. [382]

Neutrophils as a transport of diagnostic and therapeutic agents have been already demonstrated to cross the BBB and penetrate inflamed brain tumors [376]. In particular, Xue et al. used neutrophils to carry paclitaxel-loaded liposomes into the brain tumor (G422 glioma) (Fig. 13A) [377]. Unlike ordinary NPs that accumulate in tumor zones via active or passive targeting, the neutrophil-delivery system can recognize the postoperative inflammatory signals, such as IL-8 and CXCL1/KC [378], and deliver the therapeutic drugs to the infiltrating glioma. The authors demonstrated that such cell-based vehicles had a superior inhibition effect on tumor recurrence, using surgically treated glioma mouse models. Very recently, Liu et al. modified neutrophils with a lipid-decorated molecular photoacoustic contrast agent TFML. Such a cell-based delivery system demonstrated good brain tumor-targeting ability and strong photoacoustic signals in GBM [379].

Although the use of neutrophils for drug delivery is a promising strategy in the therapy of brain tumors, there are some obstacles seriously hindering this approach. First of all, there is no simple protocol to generate large numbers of neutrophils for further ex vivo injection [380]. Second, neutrophils are difficult to cultivate, and they have a very short circulatory half-life [381]. Employing the cell membrane of neutrophils for coating NPs can overcome these difficulties, even though it complicates the synthetic protocols of nanocarriers. For instance, Wang et al. developed a neutrophil-exosomes system for the delivery of antitumor drug-DOX into the glioma (Fig. 13B) [382]. The in vivo results performed on 6-Luc glioma-bearing mice models clearly showed that this cell-based vehicle can transport the drug through the BBB and migrate into the brain tumor. Zhang et al. reported a neutrophil-based microrobot that can carry cargo to the glioma in vivo [383]. The use of such a responsive system for targeted drug delivery substantially inhibits the proliferation of tumor cells compared to traditional drug injections.

#### Outlook

Currently, active work is underway to develop delivery systems for therapy of brain tumors using cell-based delivery systems [374, 384, 385]. They possess remarkable advantages such as active tumor tropism, biomimetic properties and strong immunological influence on tumor tissues. It is important to note that CAR-T cells have physiological ability to clonal expansion, which leads to a significant increase in the number of circulating antitumor cells. As a result, these cells can provide prolonged antitumor immunity, reducing the chance of relapse. Moreover, some of cell-based delivery systems are able to effectively overcome the BBB and penetrate into the membranes of the brain to reach tumor cells [367, 382, 386, 387]. However, the number of preclinical studies applying cell-based delivery systems as carriers to reach brain tumors is still limited due to the lack of understanding of mechanisms of brain tumor delivery.

## Conclusion, discussion, and prospects

Considering many methods that have been developed for overcoming the BBB to reach brain tumors, it can be concluded that the progress in the effective treatment of brain tumors is still unclear. In most cases, a lot of experimental approaches do not reach clinics, in particular, the ones related to the use of nanocarriers and cells or their derivatives. At the moment, the currently available clinical methods of brain tumor treatment are surgical intervention, chemotherapy, radiotherapy, immunotherapy, and gene therapy. Nevertheless, they cannot provide complete elimination of cancer tissues without compromising patient stability during the therapy. Moreover, there are certain side effects associated with each of these therapy methods. For example, surgical resection can damage healthy tissues of the brain that surround cancer cells. The use of chemotherapy can lead to nerve damage, nausea, hair loss, vomiting, infertility, diarrhea, insomnia and skin rash [388]. Radiotherapy causes effects mediated by exposure to ionizing radiation on healthy tissues (e.g. fibrosis, damage of the epithelial surface, radiation burn and sickness). Immunotherapy creates the risk of severe immunological reactions (e.g. systemic cytokine release syndrome) [389]. In case of gene therapy methods, inappropriate gene insertions may occur, leading to malfunctions in the activity of brain structures. The development of targeting strategies is highly required to reduce the side effects from therapy and prolong the median survival of the patients.

Nanotechnology and nanosciences have served as a major driver for the design and fabrication of various “smart” drug delivery systems with required targeting abilities [390]. The diversity of nanocarriers leads to the better understanding of the BBB penetration mechanisms and selected accumulation of the therapeutic drugs in the tumor zones. The key questions that seriously postpone the further clinical translation of the developed nanocarriers include their toxicity, reproducibility, large-scale production, and targeting ability.

(i) Toxicity: All the components that can be used for the fabrication of drug delivery systems should be non-toxic and approved by the relevant authorities. With the progress of nanotechnology, numerous new materials have been created as potential components for drug nanocarriers. However, clinical approval of new materials can be time-consuming. As a consequence, researchers focus on already known and approved materials, which limits the translation of novel ones into clinical practice.

(ii) Reproducibility: There are plenty of protocols describing the synthesis of drug delivery systems for different therapies, including brain tumors. However, these protocols are not always reproducible at the exact level as was reported, which makes further adaptation of published protocols necessary in each specific case. Automation of nanocarrier synthesis can help to overcome this problem. However, the use of complex delivery systems made of different components (organic, inorganic, and biological parts) significantly complicates the automatic synthesis process or makes it impossible.

(iii) Large-scale production: The lack of large-scale production of many modern drug delivery systems seriously limits their further translation into clinical practice. It is related to the reproducibility issues that were discussed above. Moreover, there are strict requirements for quality control, depending on the type of drug carriers, especially in the case of cell-based vehicles.

(iv) Targeting ability: Since many nanocarriers can deliver therapeutic drugs into the brain tumor via CMT, RME, and AME, the targeted delivery efficacy plays a crucial role. To enable the penetration of individual drugs and nanocarriers through the BBB, various targeting vectors can be considered for their chemical modification. However, if systemic administration is chosen, a high percentage of drug nanocarriers is accumulated in the liver and spleen. As a result, only a small fraction of drugs reaches brain tumors, significantly reducing the efficacy of therapy. After intravenous injection, nanocarriers might be covered by serum albumin, immunoglobulins, and other components of the blood, leading to their uptake by macrophages. Further, NPs can be accumulated in the spleen, lymph nodes, and liver by the reticuloendothelial system [391]. To exclude the macrophage internalization, PEGylation coating of NPs is usually performed, increasing the half-life of NPs circulation in the organism. However, in our opinion, this additional modification of NPs surface significantly complicates their further transfer into the clinical practice due to the multistep process. We suppose that the synthesis of NPs should be simple and efficient for intravenous administration with a high targeting ability. The use of cells or their derivatives can improve the efficiency of drug delivery, even though currently it remains at the initial stage [351]. There are several factors that prevent this strategy from reaching the clinics. First of all, a robust framework for ex vivo manipulation with patients’ cells should be created, which significantly complicates the therapeutic conditions and increases the final cost of this therapy. Second, the variability and time frame for the collection of biological samples required to obtain the appropriate drug delivery platform should be improved. Third, understanding of the mechanisms of cell tropism to the tumor is a key factor. Without this knowledge, it will be hard to predict the interaction between the injected cell-based vehicles and other biological objects in the live organism.

Besides, it is critical to develop combined methods of therapy. Single therapies are often insufficient for the treatment of brain tumors [7]. To improve the therapeutic efficiency, combined therapies are used, acting synergistically on different brain tumors and reducing the chance of tumor resistance. Two types of combined therapy can be distinguished. The first one is based on a simple administration of two or more compounds that have different mechanisms of action on tumors. As discussed above, brain delivery of most compounds is challenging and seems to be insufficient, according to the few clinical trials [7]. Of course, the use of appropriate targeted nanocarriers can solve this problem. But the current targeting efficiency is still too low to reach the required concentration of the delivered compounds in the brain. The second type of combined therapy suggests the use of several different treatment procedures. For instance, adjuvant therapy combines systemic chemotherapy, radiation therapy, hormone therapy, targeted therapy, biological therapy, and surgical intervention. In addition, choosing a relevant biological model is always a bottleneck in the design of new pharmaceuticals. This problem is the most common one for a wide range of cancers, especially in case of brain tumors. Thus, to predict the clinical outcome of therapeutic approach, an adequate animal model representing the complex of immunological cell-to-cell interactions in human brain tumors should be developed.

## Abbreviations

CNS: Central nervous system
WHO: World health organization
GBM: Glioblastoma
BBB: Blood–brain barrier
VLPs: Viral-like particles
BCSFB: Blood–cerebrospinal fluid barrier
CSF: Cerebrospinal fluid
RME: Receptor-mediated endocytosis
AME: Absorption-mediated endocytosis
IVI: Intracerebral ventricular injection
GLUT: D-glucose transport protein
glucose-RGD: Glucose with RGD peptide
ATP: Adenosine triphosphate
WBCs: White blood cells
PET-CT: Positron emission tomography–computed tomography
FDG-PET: Luorodeoxyglucose-positron emission tomography
L-DOPA: L-3,4-dihydroxyphenylalanine
NPs: Nanoparticles
OCTN2: Organic cation transporter
SLC22A5: Solute transporter
MAbs: Monoclonal antibodies
HIR: Human insulin receptor
IDUA: Enzyme iduronidase
VIP: Vasoactive intestinal peptide
bsAbs: Bispecific antibodies
LRP1: Receptor-related protein 1
BNCT: Boron-neutron capture therapy
PCNSL: Primary central nervous system lymphoma
BDNF: Brain-derived neurotrophic factor
TRKB: Tropomyosin receptor kinase B
P-gp: P-glycoprotein
TAT: Transcription transactivator
IGF2: Insulin-like growth factor 2
PEG: Polyethylene glycol
RV: Rabies virus
RVG: RV envelope protein glycoprotein
RV-V: RV-based vaccine
SPRAC: Secreted protein acidic and rich in cysteine
gp 60: Glycoprotein 60
TMZ: Temozolomide
HA: Hyaluronic acid
HSA: Human serum albumin
PBCA: Poly(butyl-cyanoacrylate)
QT: Quercetin
P-80: Polysorbate-80
PLA: Poly(lactic acid)
CREKA: Cysteine: arginine: glutamic acid: lysine: alanine
PLGA: Poly(lactic-co-glycolic acid)
FDA: Food drug administration
DSPC: 1,2-Distearoyl-CH-glycero-3-phosphocholine
DPFC: 1,2-Dipalmitoyl-CH-glycero-3-phosphocholine
DOPC: 1,2-Dioleoyl-snglycero-3-phosphocholine
Tf: Transferrin
Pen: Peptide-penetratin
LIFU: Low-intensity focused ultrasound
RaSP: Rapid short-pulse
SiO_2_NPs: Silica nanoparticles
Au NPs: Gold nanoparticles
IONPs: Iron oxide nanoparticles
Ag NPs: Silver nanoparticles
LSPR: Localized surface plasmon resonance
NIR: Near infrared region
RF: Radiofrequency
TEM: Transmission electron microscopy
ROS: Reactive oxygen species
PAL: Polyporus Amboinensis Lab
Abz: Albendazole
BSA: Bovine serum albumin
SPIONs: Superparamagnetic IONPs
CDs: Carbon dots
GQDs: Graphene quantum dots
CNDs: Carbon nitride dots
rBMEC: Rat brain microvascular endothelial cells
MSCs: Mesenchymal stem cells
EVs: Extracellular vesicles
SDF-1/CX: Stromal cell-derived factor-1
CXCR4: Chemokine receptor type-4
LV: Lentiviral vectors
TNF: Tumor necrosis factor
TRAIL: Tumor necrosis factor-related apoptosis-inducing ligand
BPQDs: Black phosphorus quantum dots
RBCs: Erythrocytes or red blood cells

## References

[1] “Brain, central nervous system,” 2020. https://gco.iarc.fr/today. Accessed 11 Apr 2022.

[2] Ostrom QT (2019). CBTRUS Statistical Report: Primary Brain and Other Central Nervous System Tumors Diagnosed in the United States in 2012–2016. Neuro Oncol.

[3] Howlader N, Cronin KA, Kurian AW, Andridge R (2018). Differences in breast cancer survival by molecular subtypes in the United States. Cancer Epidemiol Biomark Prev.

[4] Park EH (2017). Basic facts of breast cancer in Korea in 2014: The 10-year overall survival progress. J Breast Cancer.

[5] Višnjić A, Kovačević P, Veličkov A, Stojanović M, Mladenović S (2020). Head and neck cutaneous melanoma: 5-year survival analysis in a Serbian university center. World J Surg Oncol.

[6] Brunssen A (2020). Long-term relative survival from melanoma in Germany 1997–2013. Melanoma Res.

[7] Zhao M, van Straten D, Broekman MLD, Préat V, Schiffelers RM (2020). Nanocarrier-based drug combination therapy for glioblastoma. Theranostics.

[8] Hemminki K, Försti A, Hansson M (2021). Incidence, mortality and survival in multiple myeloma compared to other hematopoietic neoplasms in Sweden up to year 2016. Sci Rep.

[9] Quaresma M, Coleman MP, Rachet B (2015). 40-year trends in an index of survival for all cancers combined and survival adjusted for age and sex for each cancer in England and Wales, 1971–2011: a population-based study. The Lancet.

[10] Luzzi S (2019). Dysembryoplastic neuroepithelial tumors: what you need to know. World Neurosurg.

[11] Apra C, Peyre M, Kalamarides M (2018). Current treatment options for meningioma. Expert Rev Neurother.

[12] Farid M (2014). Malignant peripheral nerve sheath tumors. Oncologist.

[13] Orlova A (2018). Emerging therapeutic targets in myeloproliferative neoplasms and peripheral T-cell leukemia and lymphomas. Expert Opin Ther Targets.

[14] Bray F, Ferlay J, Soerjomataram I, Siegel RL, Torre LA, Jemal A (2018). Global cancer statistics 2018: GLOBOCAN estimates of incidence and mortality worldwide for 36 cancers in 185 countries. CA Cancer J Clin.

[15] N. Reynoso-Noverón, A. Mohar-Betancourt, J. Ortiz-Rafael. 2021. Epidemiology of Brain Tumors. In *Principles of Neuro-Oncology*, Cham: Springer International Publishing, 2021. p. 15–25. 10.1007/978-3-030-54879-7_2.

[16] Whittle IR, Smith C, Navoo P, Collie D (2004). Meningiomas. The Lancet.

[17] Ostrom QT (2014). CBTRUS statistical report: primary brain and central nervous system tumors diagnosed in the United States in 2007–2011. Neuro Oncol.

[18] Corell A (2019). Neurosurgical treatment and outcome patterns of meningioma in Sweden: a nationwide registry-based study. Acta Neurochir.

[19] Ostrom QT, Cote DJ, Ascha M, Kruchko C, Barnholtz-Sloan JS (2018). Adult glioma incidence and survival by race or ethnicity in the United States from 2000 to 2014. JAMA Oncol.

[20] Shaver M (2019). Optimizing neuro-oncology imaging: a review of deep learning approaches for glioma imaging. Cancers (Basel).

[21] Black PMCL (1991). Brain tumors. N Engl J Med.

[22] Molinaro AM, Taylor JW, Wiencke JK, Wrensch MR (2019). Genetic and molecular epidemiology of adult diffuse glioma. Nat Rev Neurol.

[23] “Survival rates for selected adult brain and spinal cord tumors.” https://www.cancer.org/cancer/brain-spinal-cord-tumors-adults/detection-diagnosis-staging/survival-rates.html (Accessed 11 Apr 2022).

[24] Fisher JL, Schwartzbaum JA, Wrensch M, Wiemels JL (2007). Epidemiology of brain tumors. Neurol Clin.

[25] Linnet K, Ejsing TB (2008). A review on the impact of P-glycoprotein on the penetration of drugs into the brain. Focus on psychotropic drugs. Eur Neuropsychopharmacol.

[26] Eyal S, Hsiao P, Unadkat JD (2009). Drug interactions at the blood-brain barrier: Fact or fantasy?☆. Pharmacol Ther.

[27] Shilo M, Motiei M, Hana P, Popovtzer R (2014). Transport of nanoparticles through the blood–brain barrier for imaging and therapeutic applications. Nanoscale.

[28] Pulicherla KK, Verma MK (2015). Targeting therapeutics across the blood brain barrier (BBB), prerequisite towards thrombolytic therapy for cerebrovascular disorders—an overview and advancements. AAPS PharmSciTech.

[29] Kadry H, Noorani B, Cucullo L (2020). A blood–brain barrier overview on structure, function, impairment, and biomarkers of integrity. Fluids Barriers CNS.

[30] Ballabh P, Braun A, Nedergaard M (2004). The blood–brain barrier: an overview. Neurobiol Dis.

[31] Liebner S, Czupalla CJ, Wolburg H (2011). Current concepts of blood-brain barrier development. Int J Dev Biol.

[32] Dong X (2018). Current strategies for brain drug delivery. Theranostics.

[33] Abbott NJ (2005). Physiology of the blood–brain barrier and its consequences for drug transport to the brain. Int Congr Ser.

[34] Jones AR, Shusta EV (2007). Blood-brain barrier transport of therapeutics via receptor-mediation”. Pharm Res.

[35] Gendelman HE (2012). The neurology of AIDS.

[36] Abbott NJ, Rönnbäck L, Hansson E (2006). Astrocyte–endothelial interactions at the blood–brain barrier. Nat Rev Neurosci.

[37] Abbott NJ, Patabendige AAK, Dolman DEM, Yusof SR, Begley DJ (2010). Structure and function of the blood–brain barrier. Neurobiol Dis.

[38] Johanson CE, Stopa EG, McMillan PN (2011). The blood-cerebrospinal fluid barrier: structure and functional significance.

[39] Engelhardt B, Sorokin L (2009). The blood–brain and the blood–cerebrospinal fluid barriers: function and dysfunction. Semin Immunopathol.

[40] Balslev Y, Saunders NR, Møllgard K (1997). Ontogenetic development of diffusional restriction to protein at the pial surface of the rat brain: an electron microscopical study. J Neurocytol.

[41] Brøchner CB, Holst CB, Møllgård K (2015). Outer brain barriers in rat and human development”. Front Neurosci.

[42] Palmer AM (2010). The role of the blood–CNS barrier in CNS disorders and their treatment. Neurobiol Dis.

[43] Dyrna F, Hanske S, Krueger M, Bechmann I (2013). The blood-brain barrier. J Neuroimmune Pharmacol.

[44] Banks WA, Erickson MA (2010). The blood–brain barrier and immune function and dysfunction. Neurobiol Dis.

[45] Gürsoy-Özdemir Y, Tas YC (2017). Anatomy and physiology of the blood-brain barrier”, in nanotechnology methods for neurological diseases and brain tumors.

[46] Dalkara T, Gursoy-Ozdemir Y, Yemisci M (2011). Brain microvascular pericytes in health and disease. Acta Neuropathol.

[47] Flügge G, Araya-Callis C, Garea-Rodriguez E, Stadelmann-Nessler C, Fuchs E (2014). NDRG2 as a marker protein for brain astrocytes. Cell Tissue Res.

[48] Korzhevskii DE, Kirik O, Sukhorukova E (2015). Immunocytochemistry of microglial cell.

[49] Hurtado-Alvarado G., Cabañas-Morales A. M., Gómez-Gónzalez B. Pericytes: brain-immune interface modulators. Frontiers in Integrative Neuroscience, vol. 7, 2014, 10.3389/fnint.2013.00080. 10.3389/fnint.2013.00080 PMC3887314 24454281

[50] Winkler EA, Bell RD, Zlokovic BV (2011). Central nervous system pericytes in health and disease. Nat Neurosci.

[51] Daneman R (2012). The blood-brain barrier in health and disease. Ann Neurol.

[52] Bradbury MW, Stubbs J, Hughes IE, Parker P (1963). THE distribution of potassium, sodium, chloride and urea between lumbar cerebrospinal fluid and blood serum in human subjects. Clin Sci.

[53] Seo S, Kim H, Sung JH, Choi N, Lee K, Kim HN (2020). Microphysiological systems for recapitulating physiology and function of blood-brain barrier. Biomaterials.

[54] Stoica R, Rusu CM, Staicu CE, Burlacu AE, Radu M, Radu BM (2021). Ca2+ homeostasis in brain microvascular endothelial cells.

[55] Hladky SB, Barrand MA (2018). Elimination of substances from the brain parenchyma: efflux via perivascular pathways and via the blood–brain barrier. Fluids Barriers CNS.

[56] Sakka L, Coll G, Chazal J (2011). Anatomy and physiology of cerebrospinal fluid. Eur Ann Otorhinolaryngol Head Neck Dis.

[57] Archie SR, al Shoyaib A, Cucullo L (2021). Blood-brain barrier dysfunction in CNS disorders and putative therapeutic targets: an overview”. Pharmaceutics.

[58] Harilal S (2020). Revisiting the blood-brain barrier: a hard nut to crack in the transportation of drug molecules. Brain Res Bull.

[59] Flanagan SD, Takahashi LH, Liu X, Benet LZ (2002). Contributions of saturable active secretion, passive transcellular, and paracellular diffusion to the overall transport of furosemide across adenocarcinoma (Caco-2) cells. J Pharm Sci.

[60] Boado RJ, Pardridge WM (2002). Glucose deprivation and hypoxia increase the expression of the GLUT1 glucose transporter via a specific mRNA cis-acting regulatory element. J Neurochem.

[61] Martins NRB (2019). Human brain/cloud interface”. Front Neurosci.

[62] A. Lalatsa and A. M. Butt. Physiology of the blood–brain barrier and mechanisms of transport across the BBB. In Nanotechnology-based targeted drug delivery systems for brain tumors. Elsevier. 2018. pp. 49–74. 10.1016/B978-0-12-812218-1.00003-8.

[63] Garg T, Bhandari S, Rath G, Goyal AK (2015). Current strategies for targeted delivery of bio-active drug molecules in the treatment of brain tumor. J Drug Target.

[64] Ohtsuki S, Terasaki T (2007). Contribution of carrier-mediated transport systems to the blood-brain barrier as a supporting and protecting interface for the brain; importance for CNS drug discovery and development. Pharm Res.

[65] Smith QR (2005). Carrier-mediated transport to enhance drug delivery to brain. Int Congr Ser.

[66] Smith MW, Gumbleton M (2006). Endocytosis at the blood–brain barrier: from basic understanding to drug delivery strategies. J Drug Target.

[67] Redzic ZB, Malatiali SA, Craik JD, Rakic ML, Isakovic AJ (2009). Blood–brain barrier efflux transport of pyrimidine nucleosides and nucleobases in the rat. Neurochem Res.

[68] W. Stillwell. Membrane transport. In An introduction to biological membranes. Elsevier. 2016. 423–451. 10.1016/B978-0-444-63772-7.00019-1.

[69] Xiao G, Gan L-S (2013). Receptor-mediated endocytosis and brain delivery of therapeutic biologics. Int J Cell Biol.

[70] Estadella I, Pedrós-Gámez O, Colomer-Molera M, Bosch M, Sorkin A, Felipe A (2020). Endocytosis: a turnover mechanism controlling ion channel function. Cells.

[71] Kaksonen M, Roux A (2018). Mechanisms of clathrin-mediated endocytosis. Nat Rev Mol Cell Biol.

[72] X. Zhu, K. Jin, Y. Huang, Z. Pang. Brain drug delivery by adsorption-mediated transcytosis. In Brain Targeted Drug Delivery System. Elsevier. 2019. p. 159–183. 10.1016/B978-0-12-814001-7.00007-X.

[73] Binder H, Lindblom G (2003). Charge-dependent translocation of the Trojan peptide penetratin across lipid membranes. Biophys J.

[74] Batrakova EV, Gendelman HE, Kabanov AV (2011). Cell-mediated drug delivery. Expert Opin Drug Deliv.

[75] Tong H-I (2016). Monocyte trafficking, engraftment, and delivery of nanoparticles and an exogenous gene into the acutely inflamed brain tissue—evaluations on monocyte-based delivery system for the central nervous system. PLoS ONE.

[76] Wahajuddin, Arora (2012). Superparamagnetic iron oxide nanoparticles: magnetic nanoplatforms as drug carriers”. Int J Nanomed.

[77] Alexander A (2019). <p>Recent expansions of novel strategies towards the drug targeting into the brain</p>. Int J Nanomed.

[78] Lipinski CA, Lombardo F, Dominy BW, Feeney PJ (1997). Experimental and computational approaches to estimate solubility and permeability in drug discovery and development settings. Adv Drug Deliv Rev.

[79] R. Tyagi, B. A. Rosa, M. Mitreva. Omics-Driven Knowledge-Based Discovery of Anthelmintic Targets and Drugs. In In Silico Drug Design. Elsevier. 2019. p. 329–358. 10.1016/B978-0-12-816125-8.00012-2.

[80] Walters WP (2012). Going further than Lipinski’s rule in drug design. Expert Opin Drug Discov.

[81] Graham CA, Cloughesy TF (2004). Brain tumor treatment: chemotherapy and other new developments. Semin Oncol Nurs.

[82] Brada M (2010). Temozolomide versus procarbazine, lomustine, and vincristine in recurrent high-grade glioma. J Clin Oncol.

[83] “Carmustine: uses, interactions, mechanism of action | drugbank online.” https://go.drugbank.com/drugs/DB00262 (Accessed 11 Apr 2022).

[84] Brandes AA, Bartolotti M, Tosoni A, Franceschi E (2016). Nitrosoureas in the management of malignant gliomas. Curr Neurol Neurosci Rep.

[85] Wang D, Wang C, Wang L, Chen Y (2019). A comprehensive review in improving delivery of small-molecule chemotherapeutic agents overcoming the blood-brain/brain tumor barriers for glioblastoma treatment. Drug Delivery.

[86] HB Newton. Clinical Pharmacology of Brain Tumor Chemotherapy. In Handbook of Brain Tumor Chemotherapy. Elsevier. 2006. p. 21–43. 10.1016/B978-012088410-0/50040-8.

[87] Marcucci F, Corti A, Ferreri AJM (2021). Breaching the blood-brain tumor barrier for tumor therapy. Cancers (Basel).

[88] Vredenburgh JJ, Desjardins A, Reardon DA, Friedman HS (2009). Experience with irinotecan for the treatment of malignant glioma. Neuro Oncol.

[89] Grommes C, DeAngelis LM (2017). Primary CNS Lymphoma. J Clin Oncol.

[90] Ferreri AJ (2009). High-dose cytarabine plus high-dose methotrexate versus high-dose methotrexate alone in patients with primary CNS lymphoma: a randomised phase 2 trial. The Lancet.

[91] Makwana V, Karanjia J, Haselhorst T, Anoopkumar-Dukie S, Rudrawar S (2021). Liposomal doxorubicin as targeted delivery platform: Current trends in surface functionalization. Int J Pharm.

[92] Warren KE (2018). Beyond the blood: brain barrier: the importance of central nervous system (CNS) pharmacokinetics for the treatment of CNS tumors, including diffuse intrinsic pontine glioma. Front Oncol.

[93] Fang F (2017). Non-invasive approaches for drug delivery to the brain based on the receptor mediated transport. Mater Sci Eng C.

[94] Siegal T (2000). In vivo assessment of the window of barrier opening after osmotic blood—brain barrier disruption in humans. J Neurosurg.

[95] Goudas LC (1999). Acute decreases in cerebrospinal fluid glutathione levels after intracerebroventricular morphine for cancer pain. Anesth Analg.

[96] Dhuria SV, Hanson LR, Frey WH (2010). Intranasal delivery to the central nervous system: mechanisms and experimental considerations. J Pharm Sci.

[97] Cereda CMS (2018). Bupivacaine in alginate and chitosan nanoparticles: an in vivo evaluation of efficacy, pharmacokinetics, and local toxicity. J Pain Res.

[98] Karashima K (2007). Prolongation of intrathecal and sciatic nerve blocks using a complex of levobupivacaine with maltosyl-β-cyclodextrin in rats. Anesth Analg.

[99] Fowler MJ, Cotter JD, Knight BE, Sevick-Muraca EM, Sandberg DI, Sirianni RW (2020). Intrathecal drug delivery in the era of nanomedicine. Adv Drug Deliv Rev.

[100] Pieretti S (2017). ‘Curcumin-loaded Poly (d, l-lactide-co-glycolide) nanovesicles induce antinociceptive effects and reduce pronociceptive cytokine and BDNF release in spinal cord after acute administration in mice’. Colloids Surf B.

[101] Cohen-Pfeffer JL (2017). Intracerebroventricular delivery as a safe, long-term route of drug administration. Pediatr Neurol.

[102] Lin J, Zhou H, Zhang N, Yin B, Sheng H (2012). Effects of the implantation of Ommaya reservoir in children with tuberculous meningitis hydrocephalus: a preliminary study. Child’s Nervous System.

[103] Wu P, Segovia D, Lee C, Nguyen K-L (2018). Consistency of continuous ambulatory interstitial glucose monitoring sensors. Biosensors (Basel).

[104] Gabathuler R (2010). Approaches to transport therapeutic drugs across the blood–brain barrier to treat brain diseases. Neurobiol Dis.

[105] Pardridge WM (2016). CSF, blood-brain barrier, and brain drug delivery. Expert Opin Drug Deliv.

[106] M Kumar, P Sharma, R Maheshwari, M Tekade, SK Shrivastava, RK Tekade. Beyond the Blood–Brain Barrier. In Nanotechnology-Based Targeted Drug Delivery Systems for Brain Tumors. Elsevier. 2018. p. 397–437. 10.1016/B978-0-12-812218-1.00015-4.

[107] Lu C-T, Zhao Y-Z, Wong HL, Cai J, Peng L, Tian X-Q (2014). Current approaches to enhance CNS delivery of drugs across the brain barriers. Int J Nanomed.

[108] Chen Q (2018). Treatment of human glioblastoma with a live attenuated zika virus vaccine candidate. mbio.

[109] Mohme M (2020). Local intracerebral immunomodulation using interleukin-expressing mesenchymal stem cells in glioblastoma. Clin Cancer Res.

[110] le Reste PJ (2020). Local intracerebral inhibition of IRE1 by MKC8866 sensitizes glioblastoma to irradiation/chemotherapy in vivo. Cancer Lett.

[111] Fleming AB, Haverstick K, Saltzman WM (2004). In vitro cytotoxicity and in vivo distribution after direct delivery of PEG−camptothecin conjugates to the rat brain. Bioconjug Chem.

[112] Yang X (2016). Peri-tumoral leakage during intra-tumoral convection-enhanced delivery has implications for efficacy of peri-tumoral infusion before removal of tumor. Drug Delivery.

[113] Ashby LS, Smith KA, Stea B (2016). Gliadel wafer implantation combined with standard radiotherapy and concurrent followed by adjuvant temozolomide for treatment of newly diagnosed high-grade glioma: a systematic literature review. World J Surg Oncol.

[114] Burkhardt J-K (2012). Intra-arterial delivery of bevacizumab after blood-brain barrier disruption for the treatment of recurrent glioblastoma: progression-free survival and overall survival. World Neurosurg.

[115] Boockvar JA (2011). Safety and maximum tolerated dose of superselective intraarterial cerebral infusion of bevacizumab after osmotic blood-brain barrier disruption for recurrent malignant glioma. J Neurosurg.

[116] Chakraborty S (2016). Superselective intraarterial cerebral infusion of cetuximab after osmotic blood/brain barrier disruption for recurrent malignant glioma: phase I study. J Neurooncol.

[117] Zylber-Katz E (2000). Pharmacokinetics of methotrexate in cerebrospinal fluid and serum after osmotic blood-brain barrier disruption in patients with brain lymphoma. Clin Pharmacol Ther.

[118] Hanson LR, Frey WH (2008). Intranasal delivery bypasses the blood-brain barrier to target therapeutic agents to the central nervous system and treat neurodegenerative disease. BMC Neurosci.

[119] Crowe TP, Greenlee MHW, Kanthasamy AG, Hsu WH (2018). Mechanism of intranasal drug delivery directly to the brain. Life Sci.

[120] Oliveira P, Fortuna A, Alves G, Falcao A (2016). Drug-metabolizing enzymes and efflux transporters in nasal epithelium: influence on the bioavailability of intranasally administered drugs. Curr Drug Metab.

[121] Bors L, Erdő F (2019). Overcoming the blood-brain barrier. challenges and tricks for CNS Drug delivery. Sci Pharm.

[122] Su Y (2020). Intranasal delivery of targeted nanoparticles loaded with miR-132 to brain for the treatment of neurodegenerative diseases. Front Pharmacol.

[123] Sabir F, Ismail R, Csoka I (2020). Nose-to-brain delivery of antiglioblastoma drugs embedded into lipid nanocarrier systems: status quo and outlook. Drug Discov Today.

[124] Zha S, Wong K, All AH (2022). Intranasal delivery of functionalized polymeric nanomaterials to the brain. Adv Healthcare Mater.

[125] Anthony DP, Hegde M, Shetty SS, Rafic T, Mutalik S, Rao BSS (2021). Targeting receptor-ligand chemistry for drug delivery across blood-brain barrier in brain diseases. Life Sci.

[126] Lockman PR, Mumper RJ, Khan MA, Allen DD (2002). Nanoparticle technology for drug delivery across the blood-brain barrier. Drug Dev Ind Pharm.

[127] Samad A, Sultana Y, Aqil M (2007). Liposomal drug delivery systems: an update review. Curr Drug Deliv.

[128] Rautio J, Gynther M, Laine K (2013). LAT1-mediated prodrug uptake: a way to breach the blood–brain barrier?. Ther Deliv.

[129] Peura L (2011). Large amino acid transporter 1 (LAT1) prodrugs of valproic acid: new prodrug design ideas for central nervous system delivery. Mol Pharm.

[130] Kageyama T (2000). The 4F2hc/LAT1 complex transports l-DOPA across the blood–brain barrier. Brain Res.

[131] Volk HA, Burkhardt K, Potschka H, Chen J, Becker A, Löscher W (2004). Neuronal expression of the drug efflux transporter P-glycoprotein in the rat hippocampus after limbic seizures. Neurosci.

[132] Kabanov AV, Batrakova EV, Miller DW (2003). Pluronic® block copolymers as modulators of drug efflux transporter activity in the blood–brain barrier. Adv Drug Deliv Rev.

[133] Jiang X (2014). Nanoparticles of 2-deoxy-d-glucose functionalized poly(ethylene glycol)-co-poly(trimethylene carbonate) for dual-targeted drug delivery in glioma treatment. Biomaterials.

[134] Pardridge WM (2007). Blood–brain barrier delivery. Drug Discov Today.

[135] Xiong F (2012). Preparation, characterization of 2-Deoxy-D-Glucose functionalized dimercaptosuccinic acid-coated maghemite nanoparticles for targeting tumor cells. Pharm Res.

[136] Ying X (2010). Dual-targeting daunorubicin liposomes improve the therapeutic efficacy of brain glioma in animals. J Control Release.

[137] Zhang Y, Qu H, Xue X (2022). Blood–brain barrier penetrating liposomes with synergistic chemotherapy for glioblastoma treatment. Biomater Sci.

[138] Li X, Qu B, Jin X, Hai L, Wu Y (2014). Design, synthesis and biological evaluation for docetaxel-loaded brain targeting liposome with ‘lock-in’ function. J Drug Target.

[139] Xie J, Shen Z, Anraku Y, Kataoka K, Chen X (2019). Nanomaterial-based blood-brain-barrier (BBB) crossing strategies. Biomaterials.

[140] Polt R (1994). Glycopeptide enkephalin analogues produce analgesia in mice: evidence for penetration of the blood-brain barrier. Proc Natl Acad Sci.

[141] Fu Q (2019). Liposomes actively recognizing the glucose transporter GLUT _1_ and integrin α _v_ β _3_ for dual-targeting of glioma. Arch Pharm (Weinheim).

[142] Liu Q (2021). Biotin and glucose co-modified multi-targeting liposomes for efficient delivery of chemotherapeutics for the treatment of glioma. Bioorg Med Chem.

[143] Schaffner P, Dard MM (2003). Structure and function of RGD peptides involved in bone biology. Cell Mol Life Sci (CMLS).

[144] Tamba BI (2018). Tailored surface silica nanoparticles for blood-brain barrier penetration: preparation and in vivo investigation. Arab J Chem.

[145] Poff AM, Ari C, Seyfried TN, D’Agostino DP (2013). The ketogenic diet and hyperbaric oxygen therapy prolong survival in mice with systemic metastatic cancer. PLoS ONE.

[146] Gillies RJ, Robey I, Gatenby RA (2008). Causes and consequences of increased glucose metabolism of cancers. J Nucl Med.

[147] Strickland M, Stoll EA (2017). Metabolic reprogramming in glioma. Front Cell Dev Biol.

[148] Poff A, Koutnik AP, Egan KM, Sahebjam S, D’Agostino D, Kumar NB (2019). Targeting the Warburg effect for cancer treatment: ketogenic diets for management of glioma. Semin Cancer Biol.

[149] Lieberman BP (2011). PET Imaging of glutaminolysis in tumors by 18 F-(2S,4R )4-fluoroglutamine. J Nucl Med.

[150] Boado RJ, Li JY, Nagaya M, Zhang C, Pardridge WM (1999). Selective expression of the large neutral amino acid transporter at the blood–brain barrier. Proc Natl Acad Sci.

[151] Geier EG (2013). Structure-based ligand discovery for the large-neutral amino acid transporter 1, LAT-1. Proc Natl Acad Sci.

[152] Li L (2016). Large amino acid transporter 1 mediated glutamate modified docetaxel-loaded liposomes for glioma targeting. Colloids Surf B.

[153] Haddad F, Sawalha M, Khawaja Y, Najjar A, Karaman R (2017). Dopamine and levodopa prodrugs for the treatment of Parkinson’s disease. Molecules.

[154] Qu M (2018). Dopamine-loaded blood exosomes targeted to brain for better treatment of Parkinson’s disease. J Control Release.

[155] Cotzias GC, van Woert MH, Schiffer LM (1967). Aromatic amino acids and modification of parkinsonism. N Engl J Med.

[156] Bhunia S (2017). Large amino acid transporter 1 selective liposomes of <scp>l</scp> -DOPA functionalized amphiphile for combating glioblastoma. Mol Pharm.

[157] Humbert O (2019). 18F-DOPA PET/CT in brain tumors: impact on multidisciplinary brain tumor board decisions. Eur J Nucl Med Mol Imaging.

[158] Capuani S (2009). Boronophenylalanine uptake in C6 glioma model is dramatically increased by l-DOPA preloading. Appl Radiat Isot.

[159] Gonzalez-Carter DA, Ong ZY, McGilvery CM, Dunlop IE, Dexter DT, Porter AE (2019). L-DOPA functionalized, multi-branched gold nanoparticles as brain-targeted nano-vehicles. Nanomed Nanotechnol Biol Med.

[160] Jones LL, McDonald DA, Borum PR (2010). Acylcarnitines: role in brain. Prog Lipid Res.

[161] Cahova M (2015). Carnitine supplementation alleviates lipid metabolism derangements and protects against oxidative stress in non-obese hereditary hypertriglyceridemic rats. Appl Physiol Nutr Metab.

[162] Zaitone SA, Abo-Elmatty DM, Shaalan AA (2012). Acetyl-l-carnitine and α-lipoic acid affect rotenone-induced damage in nigral dopaminergic neurons of rat brain, implication for Parkinson’s disease therapy. Pharmacol Biochem Behav.

[163] Virmani A, Koverech A, Ali SF, Binienda ZK (2011). Acetyl-L-carnitine modulates TP53 and IL10 gene expression induced by 3-NPA evoked toxicity in PC12 Cells. Curr Neuropharmacol.

[164] Scafidi S, Racz J, Hazelton J, McKenna MC, Fiskum G (2011). Neuroprotection by Acetyl-L-Carnitine after traumatic injury to the immature rat brain. Dev Neurosci.

[165] Kocsis K (2015). Acetyl-L-carnitine and oxaloacetate in post-treatment against LTP impairment in a rat ischemia model. An in vitro electrophysiological study. J Neural Transm.

[166] Wainwright MS, Kohli R, Whitington PF, Chace DH (2006). Carnitine treatment inhibits increases in cerebral carnitine esters and glutamate detected by mass spectrometry after hypoxia-ischemia in newborn rats. Stroke.

[167] Zhang R (2012). Neuroprotective effects of pre-treament with l-Carnitine and Acetyl-l-Carnitine on ischemic injury in vivo and in vitro. Int J Mol Sci.

[168] Juraszek B, Nałęcz KA (2019). SLC22A5 (OCTN2) Carnitine transporter—indispensable for cell metabolism, a jekyll and hyde of human cancer. Molecules.

[169] Kou L (2017). L—Carnitine-conjugated nanoparticles to promote permeation across blood–brain barrier and to target glioma cells for drug delivery via the novel organic cation/carnitine transporter OCTN2. Artificial Cells Nanomed Biotechnol.

[170] Mingorance C, Rodriguez-Rodriguez R, Justo ML, Herrera MD, de Sotomayor MA (2011). Pharmacological effects and clinical applications of propionyl-L-carnitine. Nutr Rev.

[171] Yamada S (2012). Carnitine-induced senescence in glioblastoma cells. Exp Ther Med.

[172] Randall EC (2020). Localized metabolomic gradients in patient-derived xenograft models of glioblastoma. Can Res.

[173] Han L (2019). Systemic delivery of monoclonal antibodies to the central nervous system for brain tumor therapy. Adv Mater.

[174] Zeiadeh I, Najjar A, Karaman R (2018). Strategies for enhancing the permeation of CNS-active drugs through the blood-brain barrier: a review. Molecules.

[175] Pardridge W (2006). Molecular Trojan horses for blood–brain barrier drug delivery. Curr Opin Pharmacol.

[176] Sousa F, Moura RP, Moreira E, Martins C, Sarmento B (2018). “Therapeutic monoclonal antibodies delivery for the glioblastoma treatment.

[177] Pardridge WM, Kang Y, Buciak JL, Yang J (1995). Human insulin receptor monoclonal antibody undergoes high affinity binding to human brain capillaries in vitro and rapid transcytosis through the blood-brain barrier in vivo in the primate. Pharm Res.

[178] Wu D, Yang J, Pardridge WM (1997). Drug targeting of a peptide radiopharmaceutical through the primate blood-brain barrier in vivo with a monoclonal antibody to the human insulin receptor. J Clin Investig.

[179] Sumbria RK, Boado RJ, Pardridge WM (2012). Imaging amyloid plaque in Alzheimer’s disease brain with a biotinylated Aβ peptide radiopharmaceutical conjugated to an IgG-Avidin fusion protein. Bioconjug Chem.

[180] Boado RJ, Lu JZ, Hui EK-W, Sumbria RK, Pardridge WM (2013). Pharmacokinetics and brain uptake in the rhesus monkey of a fusion protein of arylsulfatase a and a monoclonal antibody against the human insulin receptor. Biotechnol Bioeng.

[181] Lee HJ, Engelhardt B, Lesley J, Bickel U, Pardridge WM (2000). Targeting rat anti-mouse transferrin receptor monoclonal antibodies through blood-brain barrier in mouse. J Pharmacol Exp Ther.

[182] Bickel U, Yoshikawa T, Landaw EM, Faull KF, Pardridge WM (1993). Pharmacologic effects in vivo in brain by vector-mediated peptide drug delivery. Proc Natl Acad Sci.

[183] Moody TW, Nuche-Berenguer B, Jensen RT (2016). Vasoactive intestinal peptide/pituitary adenylate cyclase activating polypeptide, and their receptors and cancer. Curr Opin Endocrinol Diabetes Obes.

[184] Khan AR, Liu M, Khan MW, Zhai G (2017). Progress in brain targeting drug delivery system by nasal route. J Control Release.

[185] Li T (2016). Camelid single-domain antibodies: a versatile tool for in vivo imaging of extracellular and intracellular brain targets. J Control Release.

[186] Sui Y-T, Bullock KM, Erickson MA, Zhang J, Banks WA (2014). Alpha synuclein is transported into and out of the brain by the blood–brain barrier. Peptides.

[187] Benchenane K (2005). Tissue-type plasminogen activator crosses the intact blood-brain barrier by low-density lipoprotein receptor-related protein-mediated transcytosis. Circulation.

[188] Yang W (2008). Molecular targeting and treatment of composite EGFR and EGFRvIII-positive gliomas using boronated monoclonal antibodies. Clin Cancer Res.

[189] Lampson LA (2011). Monoclonal antibodies in neuro-oncology. MAbs.

[190] Galstyan A (2019). Blood–brain barrier permeable nano immunoconjugates induce local immune responses for glioma therapy. Nat Commun.

[191] Gan HK, van den Bent M, Lassman AB, Reardon DA, Scott AM (2017). Antibody–drug conjugates in glioblastoma therapy: the right drugs to the right cells. Nat Rev Clin Oncol.

[192] Wu D, Pardridge WM (1999). Neuroprotection with noninvasive neurotrophin delivery to the brain. Proc Natl Acad Sci.

[193] Zhang Y, Pardridge WM (2001). Neuroprotection in transient focal brain ischemia after delayed intravenous administration of brain-derived neurotrophic factor conjugated to a blood-brain barrier drug targeting system. Stroke.

[194] Pardridge WM (2017). Delivery of biologics across the blood-brain barrier with molecular Trojan Horse technology. BioDrugs.

[195] Oldendorf WH (1981). “Blood-brain barrier permeability to peptides: pitfalls in measurement. Peptides.

[196] Temsamani J, Vidal P (2004). The use of cell-penetrating peptides for drug delivery. Drug Discov Today.

[197] Meisenberg G, Simmons WH (1983). Peptides and the blood-brain barrier. Life Sci.

[198] Rousselle C, Clair P, Lefauconnier J-M, Kaczorek M, Scherrmann J-M, Temsamani J (2000). New advances in the transport of Doxorubicin through the blood-brain barrier by a peptide vector-mediated strategy. Mol Pharmacol.

[199] Seth AK, Barrett AB, Barnett L (2015). Granger causality analysis in neuroscience and neuroimaging. J Neurosci.

[200] Sarko D (2010). The pharmacokinetics of cell-penetrating peptides. Mol Pharm.

[201] Kan S (2014). Delivery of an enzyme-IGFII fusion protein to the mouse brain is therapeutic for mucopolysaccharidosis type IIIB. Proc Natl Acad Sci.

[202] Wanjale MV, Kumar GSV (2017). Peptides as a therapeutic avenue for nanocarrier-aided targeting of glioma. Expert Opin Drug Deliv.

[203] Yao H (2015). Enhanced blood–brain barrier penetration and glioma therapy mediated by a new peptide modified gene delivery system. Biomaterials.

[204] Chen L (2019). Blood–brain barrier- and blood-brain tumor barrier-penetrating peptide-derived targeted therapeutics for glioma and malignant tumor brain metastases. ACS Appl Mater Interfaces.

[205] Sánchez-Navarro M, Giralt E, Teixidó M (2017). Blood–brain barrier peptide shuttles. Curr Opin Chem Biol.

[206] Laksitorini MD, Kiptoo PK, On NH, Thliveris JA, Miller DW, Siahaan TJ (2015). Modulation of intercellular junctions by cyclic-ADT peptides as a method to reversibly increase blood-brain barrier permeability. J Pharm Sci.

[207] Régina A (2008). Antitumour activity of ANG1005, a conjugate between paclitaxel and the new brain delivery vector angiopep-2. Br J Pharmacol.

[208] Demeule M (2008). Involvement of the low-density lipoprotein receptor-related protein in the transcytosis of the brain delivery vector angiopep-2. J Neurochem.

[209] Mashel TV (2020). Overcoming the delivery problem for therapeutic genome editing: Current status and perspective of non-viral methods. Biomaterials.

[210] Meyers JD, Doane T, Burda C, Basilion JP (2013). Nanoparticles for imaging and treating brain cancer. Nanomedicine.

[211] Timin AS, Litvak MM, Gorin DA, Atochina-Vasserman EN, Atochin DN, Sukhorukov GB (2018). Cell-based drug delivery and use of nano-and microcarriers for cell functionalization. Adv Healthcare Mater.

[212] Manikandan C, Kaushik A, Sen D (2020). Viral vector: potential therapeutic for glioblastoma multiforme. Cancer Gene Ther.

[213] Mohsen MO, Speiser DE, Knuth A, Bachmann MF (2020). Virus-like particles for vaccination against cancer. WIREs Nanomed Nanobiotechnol.

[214] Draz MS, Shafiee H (2018). Applications of gold nanoparticles in virus detection. Theranostics.

[215] Bacteriophage Virus-Like Particles as a Platform for Vaccine Discovery. In Viral Nanotechnology, CRC Press. 2015. p. 260–271. 10.1002/wnan.119.

[216] Mahoney DJ, Stojdl DF, Laird G (2014). Virus therapy for cancer. Sci Am.

[217] Gao Y (2014). RVG-Peptide-linked trimethylated chitosan for delivery of siRNA to the brain. Biomacromol.

[218] Ugolini G (2010). Advances in viral transneuronal tracing. J Neurosci Methods.

[219] Altinoz MA, Guloksuz S, Elmaci İ (2017). Rabies virus vaccine as an immune adjuvant against cancers and glioblastoma: new studies may resurrect a neglected potential. Clin Transl Oncol.

[220] Hooper DC, Roy A, Kean RB, Phares TW, Barkhouse DA (2011). Therapeutic immune clearance of rabies virus from the CNS. Futur Virol.

[221] Lebrun A, Portocarrero C, Kean RB, Barkhouse DA, Faber M, Hooper DC (2015). T-bet is required for the rapid clearance of attenuated rabies virus from central nervous system tissue. J Immunol.

[222] Philipov PV. Adjuvant treatment of brain glioblastoma multiforme with rabies vaccine, deferoxamine and dpenicillamine: a pilot study. J Biomed Clin Res. 2009;2(1):49-53

[223] E Bongiorno, M Morin-Brureau, D Barkhouse, M Faber, B Dietzschold, D Hooper. A novel rabies virus-based glioma immunotherapeutic strategy targeting survivin (P4440). J Immunol. 2013. 190(Suppl 1).

[224] Liao Z, Tu L, Li X, Liang X-J, Huo S (2021). Virus-inspired nanosystems for drug delivery. Nanoscale.

[225] Ferrer-Miralles N, Rodríguez-Carmona E, Corchero JL, García-Fruitós E, Vázquez E, Villaverde A (2015). Engineering protein self-assembling in protein-based nanomedicines for drug delivery and gene therapy. Crit Rev Biotechnol.

[226] Lu Y, Chan W, Ko BY, VanLang CC, Swartz JR (2015). Assessing sequence plasticity of a virus-like nanoparticle by evolution toward a versatile scaffold for vaccines and drug delivery. Proc Natl Acad Sci.

[227] Zuber G (2001). Towards synthetic viruses. Adv Drug Deliv Rev.

[228] Pang H-H (2019). Convection-enhanced delivery of a virus-like nanotherapeutic agent with dual-modal imaging for besiegement and eradication of brain tumors. Theranostics.

[229] Yang J (2020). Nanoparticle-based co-delivery of siRNA and paclitaxel for dual-targeting of glioblastoma. Nanomedicine.

[230] Hefferon K (2016). Plant virus nanoparticles: new applications and new benefits. Futur Virol.

[231] Martínez-Molina E, Chocarro-Wrona C, Martínez-Moreno D, Marchal JA, Boulaiz H (2020). Large-scale production of lentiviral vectors: current perspectives and challenges. Pharmaceutics.

[232] Grein TA, Weidner T, Czermak P (2017). Concepts for the production of viruses and viral vectors in cell cultures. New Insights into Cell Culture Technol INTECH.

[233] A Roldão, AC Silva, MCM Mellado, PM Alves, MJT Carrondo, Viruses and Virus-Like Particles in Biotechnology: Fundamentals and Applications. In Comprehensive biotechnology. Elsevier. 2017. p. 633–656. 10.1016/B978-0-12-809633-8.09046-4.

[234] Le DT, Müller KM (2021). In vitro assembly of virus-like particles and their applications. Life.

[235] Nooraei S (2021). Virus-like particles: preparation, immunogenicity and their roles as nanovaccines and drug nanocarriers. J Nanobiotechnol.

[236] Jena L, McErlean E, McCarthy H (2020). Delivery across the blood-brain barrier: nanomedicine for glioblastoma multiforme. Drug Deliv Transl Res.

[237] Lin T (2016). Blood–brain-barrier-penetrating albumin nanoparticles for biomimetic drug delivery *via* albumin-binding protein pathways for antiglioma therapy. ACS Nano.

[238] Gregory JV (2020). Systemic brain tumor delivery of synthetic protein nanoparticles for glioblastoma therapy. Nat Commun.

[239] Kudarha RR, Sawant KK (2021). Hyaluronic acid conjugated albumin nanoparticles for efficient receptor mediated brain targeted delivery of temozolomide. J Drug Deliv Sci Technol.

[240] Kreuter J, Alyautdin RN, Kharkevich DA, Ivanov AA (1995). Passage of peptides through the blood-brain barrier with colloidal polymer particles (nanoparticles). Brain Res.

[241] Weiss CK (2008). The first step into the brain: uptake of NIO-PBCA nanoparticles by endothelial cells in vitro and in vivo, and direct evidence for their blood-brain barrier permeation. Chem Med Chem.

[242] Zhang Y, Lin S, Wang X, Zhu G (2019). Nanovaccines for cancer immunotherapy. WIREs Nanomed Nanobiotechnol.

[243] Voigt N, Henrich-Noack P, Kockentiedt S, Hintz W, Tomas J, Sabel BA (2014). Toxicity of polymeric nanoparticles in vivo and in vitro. J Nanopart Res.

[244] Khan Z, Bagad M (2015). Poly(n-butylcyanoacrylate) nanoparticles for oral delivery of quercetin: preparation, characterization, and pharmacokinetics and biodistribution studies in Wistar rats. Int J Nanomed.

[245] Pang Z (2014). Polyethylene glycol-polylactic acid nanoparticles modified with cysteine-arginine-glutamic acid-lysine-alanine fibrin-homing peptide for glioblastoma therapy by enhanced retention effect. Int J Nanomed.

[246] Kang T (2016). Synergistic targeting tenascin C and neuropilin-1 for specific penetration of nanoparticles for anti-glioblastoma treatment. Biomaterials.

[247] Seo Y-E (2019). Nanoparticle-mediated intratumoral inhibition of miR-21 for improved survival in glioblastoma. Biomaterials.

[248] Madani F (2020). Paclitaxel/methotrexate co-loaded PLGA nanoparticles in glioblastoma treatment: formulation development and in vitro antitumor activity evaluation. Life Sci.

[249] Ganipineni LP (2018). Magnetic targeting of paclitaxel-loaded poly(lactic-<em>co</em>-glycolic acid)-based nanoparticles for the treatment of glioblastoma. Int J Nanomed.

[250] Caban-Toktas S (2020). Combination of paclitaxel and R-flurbiprofen loaded PLGA nanoparticles suppresses glioblastoma growth on systemic administration. Int J Pharm.

[251] Mohanta BC (2019). Lipid based nanoparticles: current strategies for brain tumor targeting. Current Nanomater.

[252] Guimarães D, Cavaco-Paulo A, Nogueira E (2021). Design of liposomes as drug delivery system for therapeutic applications. Int J Pharm.

[253] Beltrán-Gracia E, López-Camacho A, Higuera-Ciapara I, Velázquez-Fernández JB, Vallejo-Cardona AA (2019). Nanomedicine review: clinical developments in liposomal applications. Cancer Nanotechnol.

[254] Craig DQM, Taylor KMG, Barker SA (2011). Calorimetric investigations of liposome formation. J Pharm Pharmacol.

[255] Manconi M, Mura S, Sinico C, Fadda AM, Vila AO, Molina F (2009). Development and characterization of liposomes containing glycols as carriers for diclofenac. Colloids Surf A.

[256] Frézard F (1999). Liposomes: from biophysics to the design of peptide vaccines. Braz J Med Biol Res.

[257] Ghadiri M, Vasheghani-Farahani E, Atyabi F, Kobarfard F, Mohamadyar-Toupkanlou F, Hosseinkhani H (2017). Transferrin-conjugated magnetic dextran-spermine nanoparticles for targeted drug transport across blood-brain barrier. J Biomed Mater Res Part A.

[258] Gregori M (2016). Novel antitransferrin receptor antibodies improve the blood-brain barrier crossing efficacy of immunoliposomes. J Pharm Sci.

[259] Kim K-T (2018). Nanodelivery systems for overcoming limited transportation of therapeutic molecules through the blood–brain barrier. Future Med Chem.

[260] Razpotnik R, Novak N, Čurin Šerbec V, Rajcevic U (2017). Targeting malignant brain tumors with antibodies. Front Immunol.

[261] Song S (2008). Peptide ligand-mediated liposome distribution and targeting to EGFR expressing tumor in vivo. Int J Pharm.

[262] Jhaveri A, Deshpande P, Pattni B, Torchilin V (2018). Transferrin-targeted, resveratrol-loaded liposomes for the treatment of glioblastoma. J Control Release.

[263] Wang X (2019). Cell-penetrating peptide and transferrin co-modified liposomes for targeted therapy of glioma. Molecules.

[264] Zong T (2014). Synergistic dual-ligand doxorubicin liposomes improve targeting and therapeutic efficacy of brain glioma in animals. Mol Pharm.

[265] Beier CP (2009). RNOP-09: pegylated liposomal doxorubicine and prolonged temozolomide in addition to radiotherapy in newly diagnosed glioblastoma—a phase II study. BMC Cancer.

[266] Lakkadwala S, dos Rodrigues BS, Sun C, Singh J (2019). Dual functionalized liposomes for efficient co-delivery of anti-cancer chemotherapeutics for the treatment of glioblastoma. J Controll Release.

[267] Choi JJ, Pernot M, Small SA, Konofagou EE (2007). Noninvasive, transcranial and localized opening of the blood-brain barrier using focused ultrasound in mice. Ultrasound Med Biol.

[268] O’Reilly MA, Waspe AC, Ganguly M, Hynynen K (2011). Focused-ultrasound disruption of the blood-brain barrier using closely-timed short pulses: influence of sonication parameters and injection rate. Ultrasound Med Biol.

[269] Lin Q (2016). Brain tumor-targeted delivery and therapy by focused ultrasound introduced doxorubicin-loaded cationic liposomes. Cancer Chemother Pharmacol.

[270] Papachristodoulou A (2019). Chemotherapy sensitization of glioblastoma by focused ultrasound-mediated delivery of therapeutic liposomes. J Control Release.

[271] Morse SV, Mishra A, Chan TG, de Rosales RTM, Choi JJ (2022). Liposome delivery to the brain with rapid short-pulses of focused ultrasound and microbubbles. J Controlled Release.

[272] Khorsandi K, Hosseinzadeh R, Sadat Esfahani H, Keyvani-Ghamsari S, Ur Rahman S (2021). Nanomaterials as drug delivery systems with antibacterial properties: current trends and future priorities. Expert Rev Anti-infect Ther.

[273] Hussein Kamareddine M (2019). Organic nanoparticles as drug delivery systems and their potential role in the treatment of chronic myeloid leukemia. Technol Cancer Res Treat.

[274] Jo DH, Kim JH, Lee TG, Kim JH (2015). Size, surface charge, and shape determine therapeutic effects of nanoparticles on brain and retinal diseases. Nanomed Nanotechnol Biol Med.

[275] Lei C, Davoodi P, Zhan W, Chow PK-H, Wang C-H (2019). Development of nanoparticles for drug delivery to brain tumor: the effect of surface materials on penetration into brain tissue. J Pharm Sci.

[276] Chan M-H, Chang Z-X, Huang C-YF, Lee LJ, Liu R-S, Hsiao M (2022). Integrated therapy platform of exosomal system: hybrid inorganic/organic nanoparticles with exosomes for cancer treatment. Nanoscale Horizons.

[277] Janjua TI, Cao Y, Yu C, Popat A (2021). Clinical translation of silica nanoparticles. Nat Rev Mater.

[278] Yang Y, Zhang M, Song H, Yu C (2020). Silica-based nanoparticles for biomedical applications: from nanocarriers to biomodulators. Acc Chem Res.

[279] Turan O (2019). Delivery of drugs into brain tumors using multicomponent silica nanoparticles. Nanoscale.

[280] Juthani R (2020). Ultrasmall core-shell silica nanoparticles for precision drug delivery in a high-grade malignant brain tumor model. Clin Cancer Res.

[281] Zhu J (2021). Angiopep-2 modified lipid-coated mesoporous silica nanoparticles for glioma targeting therapy overcoming BBB. Biochem Biophys Res Commun.

[282] Heggannavar GB, Vijeth S, Kariduraganavar MY (2019). Development of dual drug loaded PLGA based mesoporous silica nanoparticles and their conjugation with Angiopep-2 to treat glioma. J Drug Deliv Sci Technol.

[283] Ma N, Liu P, He N, Gu N, Wu F-G, Chen Z (2017). Action of gold nanospikes-based nanoradiosensitizers: cellular internalization, radiotherapy, and autophagy. ACS Appl Mater Interfaces.

[284] Her S, Jaffray DA, Allen C (2017). Gold nanoparticles for applications in cancer radiotherapy: mechanisms and recent advancements. Adv Drug Deliv Rev.

[285] Li J (2021). Near infrared photothermal conversion materials: mechanism, preparation, and photothermal cancer therapy applications. J Mater Chem B.

[286] Cole LE, Ross RD, Tilley JM, Vargo-Gogola T, Roeder RK (2015). Gold nanoparticles as contrast agents in x-ray imaging and computed tomography. Nanomedicine.

[287] Norouzi M (2020). Gold nanoparticles in glioma theranostics. Pharmacol Res.

[288] Meola A, Rao J, Chaudhary N, Sharma M, Chang SD (2018). Gold nanoparticles for brain tumor imaging: a systematic review. Front Neurol.

[289] Coluccia D (2018). Enhancing glioblastoma treatment using cisplatin-gold-nanoparticle conjugates and targeted delivery with magnetic resonance-guided focused ultrasound. Nanomed Nanotechnol Biol Med.

[290] Dong C (2021). Multifunctionalized gold sub-nanometer particles for sensitizing radiotherapy against glioblastoma. Small.

[291] Amini SM, Mahabadi VP (2018). Selenium nanoparticles role in organ systems functionality and disorder. Nanomed Res J.

[292] Li S, Bian F, Yue L, Jin H, Hong Z, Shu G (2014). Selenium-dependent antitumor immunomodulating activity of polysaccharides from roots of A. membranaceus. Int J Biol Macromol.

[293] Park SO (2015). Effects of combination therapy of docetaxel with selenium on the human breast cancer cell lines MDA-MB-231 and MCF-7. Annals Surg Treat Res.

[294] Guo L, Huang K, Liu H (2016). Biocompatibility selenium nanoparticles with an intrinsic oxidase-like activity. J Nanopart Res.

[295] Gong G (2018). Targeted delivery of paclitaxel by functionalized selenium nanoparticles for anticancer therapy through ROS-mediated signaling pathways. RSC Adv.

[296] Xu B (2020). Selenium nanoparticles reduce glucose metabolism and promote apoptosis of glioma cells through reactive oxygen species-dependent manner. NeuroReport.

[297] Sonkusre P, Cameotra SS (2017). Biogenic selenium nanoparticles induce ROS-mediated necroptosis in PC-3 cancer cells through TNF activation. J Nanobiotechnol.

[298] Chen M, Huang Y, Zhu X, Hu X, Chen T (2018). Efficient overcoming of blood-brain barrier by functionalized selenium nanoparticles to treat glioma. Adv Ther.

[299] Song Z, Liu T, Chen T (2018). Overcoming blood–brain barrier by HER2-targeted nanosystem to suppress glioblastoma cell migration, invasion and tumor growth. J Mater Chem B.

[300] Liu W (2021). RGD peptide-conjugated selenium nanocomposite inhibits human glioma growth by triggering mitochondrial dysfunction and ROS-dependent MAPKs activation. Front Bioeng Biotechnol.

[301] Báez DF, Gallardo-Toledo E, Oyarzún MP, Araya E, Kogan MJ (2021). The influence of size and chemical composition of silver and gold nanoparticles on in vivo toxicity with potential applications to central nervous system diseases. Int J Nanomed.

[302] Koryakina I, Kuznetsova DS, Zuev DA, Milichko VA, Timin AS, Zyuzin MV (2020). Optically responsive delivery platforms: from the design considerations to biomedical applications. Nanophotonics.

[303] Bao Z, Liu X, Liu Y, Liu H, Zhao K (2016). Near-infrared light-responsive inorganic nanomaterials for photothermal therapy. Asian J Pharm Sci.

[304] Park T (2020). ICG-loaded PEGylated BSA-silver nanoparticles for effective photothermal cancer therapy. Int J Nanomed.

[305] Urbańska K (2015). The effect of silver nanoparticles (AgNPs) on proliferation and apoptosis of in ovo cultured glioblastoma multiforme (GBM) cells. Nanoscale Res Lett.

[306] Zhang Y, Lu H, Yu D, Zhao D (2017). AgNPs and Ag/C225 exert anticancerous effects via cell cycle regulation and cytotoxicity enhancement. J Nanomater.

[307] Yang T, Yao Q, Cao F, Liu Q, Liu B, Wang X (2016). Silver nanoparticles inhibit the function of hypoxia-inducible factor-1 and target genes: insight into the cytotoxicity and antiangiogenesis. Int J Nanomed.

[308] AshaRani P, Hande MP, Valiyaveettil S (2009). Anti-proliferative activity of silver nanoparticles. BMC Cell Biol.

[309] Moutin M-J, Abramson JJ, Salama G, Dupont Y (1989). Rapid Ag+-induced release of Ca2+ from sarcoplasmic reticulum vesicles of skeletal muscle: a rapid filtration study. Biochim Biophys Acta.

[310] Liang J (2019). Menthol-modified BSA nanoparticles for glioma targeting therapy using an energy restriction strategy. NPG Asia Mater.

[311] Dan M (2015). Silver nanoparticles induce tight junction disruption and astrocyte neurotoxicity in a rat blood&ndash;brain barrier primary triple coculture model. Int J Nanomed.

[312] Xu L, Wang Y-Y, Huang J, Chen C-Y, Wang Z-X, Xie H (2020). Silver nanoparticles: synthesis, medical applications and biosafety. Theranostics.

[313] Chen J, Yuan M, Madison CA, Eitan S, Wang Y (2022). Blood-brain barrier crossing using magnetic stimulated nanoparticles. J Control Release.

[314] Zyuzin MV (2019). Confining iron oxide nanocubes inside submicrometric cavities as a key strategy to preserve magnetic heat losses in an intracellular environment. ACS Appl Mater Interfaces.

[315] Liu H (2016). Application of iron oxide nanoparticles in glioma imaging and therapy: from bench to bedside. Nanoscale.

[316] Bustamante-Torres M, Romero-Fierro D, Estrella-Nuñez J, Arcentales-Vera B, Chichande-Proaño E, Bucio E (2022). Polymeric composite of magnetite iron oxide nanoparticles and their application in biomedicine: a review. Polymers (Basel).

[317] Wu Y, Lu Z, Li Y, Yang J, Zhang X (2020). Surface modification of iron oxide-based magnetic nanoparticles for cerebral theranostics: application and prospection. Nanomaterials.

[318] Cortés-Llanos B (2021). Influence of coating and size of magnetic nanoparticles on cellular uptake for in vitro MRI. Nanomaterials.

[319] Qiu Y (2017). Magnetic forces enable controlled drug delivery by disrupting endothelial cell-cell junctions. Nat Commun.

[320] Huang Y, Zhang B, Xie S, Yang B, Xu Q, Tan J (2016). Superparamagnetic iron oxide nanoparticles modified with tween 80 pass through the intact blood-brain barrier in rats under magnetic field. ACS Appl Mater Interfaces.

[321] Wadajkar AS (2021). Surface-modified nanodrug carriers for brain cancer treatment.

[322] Chen G-J (2014). Angiopep-pluronic F127-conjugated superparamagnetic iron oxide nanoparticles as nanotheranostic agents for BBB targeting. J Mater Chem B.

[323] Tan J (2019). I6P7 peptide modified superparamagnetic iron oxide nanoparticles for magnetic resonance imaging detection of low-grade brain gliomas. J Mater Chem B.

[324] Tabatabaei SN, Girouard H, Carret A-S, Martel S (2015). Remote control of the permeability of the blood–brain barrier by magnetic heating of nanoparticles: a proof of concept for brain drug delivery. J Control Release.

[325] Lammers T (2015). Theranostic USPIO-Loaded microbubbles for mediating and monitoring blood-brain barrier permeation. Adv Func Mater.

[326] Wu VM, Huynh E, Tang S, Uskoković V (2019). Brain and bone cancer targeting by a ferrofluid composed of superparamagnetic iron-oxide/silica/carbon nanoparticles (earthicles). Acta Biomater.

[327] Zhang W (2021). Carbon dots: a future blood-brain barrier penetrating nanomedicine and drug nanocarrier. Int J Nanomed.

[328] Tejwan N, Saha SK, Das J (2020). Multifaceted applications of green carbon dots synthesized from renewable sources. Adv Coll Interface Sci.

[329] Mintz KJ, Zhou Y, Leblanc RM (2019). Recent development of carbon quantum dots regarding their optical properties, photoluminescence mechanism, and core structure. Nanoscale.

[330] Shi X (2019). Far-red to near-infrared carbon dots: preparation and applications in biotechnology. Small.

[331] Liyanage PY (2019). Carbon nitride dots: a selective bioimaging nanomaterial. Bioconjug Chem.

[332] Lu S (2016). Hydrothermal synthesis of nitrogen-doped carbon dots with real-time live-cell imaging and blood&ndash;brain barrier penetration capabilities. Int J Nanomed.

[333] Niu Y (2020). Protein–carbon dot nanohybrid-based early blood-brain barrier damage theranostics. ACS Appl Mater Interfaces.

[334] Alam Bony B, Kievit FM (2019). A role for nanoparticles in treating traumatic brain injury. Pharmaceutics.

[335] Hettiarachchi SD (2019). Triple conjugated carbon dots as a nano-drug delivery model for glioblastoma brain tumors. Nanoscale.

[336] Qiao L, Sun T, Zheng X, Zheng M, Xie Z (2018). Exploring the optimal ratio of d-glucose/l-aspartic acid for targeting carbon dots toward brain tumor cells. Mater Sci Eng, C.

[337] Li J (2021). Exosome-coated ^10^ B carbon dots for precise boron neutron capture therapy in a mouse model of glioma in situ. Adv Func Mater.

[338] Imran M, Ahmed AAA, Kateb B, Kaushik A (2021). Inorganic Nanostructures for Brain Tumor Management.

[339] Huang H-C, Barua S, Sharma G, Dey SK, Rege K (2011). Inorganic nanoparticles for cancer imaging and therapy. J Control Release.

[340] Qiao R (2012). Receptor-mediated delivery of magnetic nanoparticles across the blood-brain barrier. ACS Nano.

[341] Thomsen LB, Thomsen MS, Moos T (2015). Targeted drug delivery to the brain using magnetic nanoparticles. Ther Deliv.

[342] Teleanu D, Chircov C, Grumezescu A, Volceanov A, Teleanu R (2018). Impact of nanoparticles on brain health: an up to date overview. J Clin Med.

[343] Ealia SAM, Saravanakumar MP (2017). A review on the classification, characterisation, synthesis of nanoparticles and their application. IOP Conf Series Mater Sci Eng.

[344] N Strambeanu, L Demetrovici, D Dragos, M Lungu, Nanoparticles: definition, classification and general physical properties. In Nanoparticles’ promises and risks, Cham: Springer International Publishing. 2015. p. 3–8. 10.1007/978-3-319-11728-7_1.

[345] Santos T (2014). Sequential administration of carbon nanotubes and near-infrared radiation for the treatment of gliomas. Front Oncol.

[346] Nam J (2013). Surface engineering of inorganic nanoparticles for imaging and therapy. Adv Drug Deliv Rev.

[347] Cho EC, Glaus C, Chen J, Welch MJ, Xia Y (2010). Inorganic nanoparticle-based contrast agents for molecular imaging. Trends Mol Med.

[348] Chen J (2005). Gold nanocages: bioconjugation and their potential use as optical imaging contrast agents. Nano Lett.

[349] Popovtzer R (2008). Targeted gold nanoparticles enable molecular CT imaging of cancer. Nano Lett.

[350] Sun C, Lee JSH, Zhang M (2008). Magnetic nanoparticles in MR imaging and drug delivery. Adv Drug Deliv Rev.

[351] Mendanha D, Vieira de Castro J, Ferreira H, Neves NM (2021). Biomimetic and cell-based nanocarriers—new strategies for brain tumor targeting. J Control Release.

[352] Young JS, Kim JW, Ahmed AU, Lesniak MS (2014). Therapeutic cell carriers: a potential road to cure glioma. Expert Rev Neurother.

[353] Li Y-J (2021). From blood to brain: blood cell-based biomimetic drug delivery systems. Drug Delivery.

[354] Porada CD, Rodman C, Ignacio G, Atala A, Almeida-Porada G (2014). Hemophilia A: an ideal disease to correct in utero. Front Pharmacol.

[355] Pal B, Das B (2017). In vitro culture of naïve human bone marrow mesenchymal stem cells: a stemness based approach. Front Cell Dev Biol.

[356] Phi LTH (2018). Cancer stem cells (CSCs) in drug resistance and their therapeutic implications in cancer treatment. Stem Cells Int.

[357] Baraniak PR, McDevitt TC (2010). Stem cell paracrine actions and tissue regeneration. Regen Med.

[358] Nieddu V (2019). Engineered human mesenchymal stem cells for neuroblastoma therapeutics. Oncol Rep.

[359] Roger M (2010). Mesenchymal stem cells as cellular vehicles for delivery of nanoparticles to brain tumors. Biomaterials.

[360] Timin AS (2019). Safe and effective delivery of antitumor drug using mesenchymal stem cells impregnated with submicron carriers. ACS Appl Mater Interfaces.

[361] Muslimov AR (2020). Biomimetic drug delivery platforms based on mesenchymal stem cells impregnated with light-responsive submicron sized carriers. Biomater Sci.

[362] Luo M (2020). Mesenchymal stem cells transporting black phosphorus-based biocompatible nanospheres: active trojan horse for enhanced photothermal cancer therapy. Chem Eng J.

[363] Su Y, Xie Z, Kim GB, Dong C, Yang J (2015). Design strategies and applications of circulating cell-mediated drug delivery systems. ACS Biomater Sci Eng.

[364] Fu S (2019). Dual-modified novel biomimetic nanocarriers improve targeting and therapeutic efficacy in glioma. ACS Appl Mater Interfaces.

[365] Chai Z (2017). A facile approach to functionalizing cell membrane-coated nanoparticles with neurotoxin-derived peptide for brain-targeted drug delivery. J Control Release.

[366] Cui Y (2020). Dual-target peptide-modified erythrocyte membrane-enveloped PLGA nanoparticles for the treatment of glioma. Front Oncol.

[367] Chai Z (2019). Ligand-modified cell membrane enables the targeted delivery of drug nanocrystals to glioma. ACS Nano.

[368] Lim M, Xia Y, Bettegowda C, Weller M (2018). Current state of immunotherapy for glioblastoma. Nat Rev Clin Oncol.

[369] Jiang W (2017). Designing nanomedicine for immuno-oncology. Nat Biomed Eng.

[370] Akhavan D, Alizadeh D, Wang D, Weist MR, Shepphird JK, Brown CE (2019). CAR T cells for brain tumors: Lessons learned and road ahead. Immunol Rev.

[371] Brown CE (2018). Optimization of IL13Rα2-targeted chimeric antigen receptor T cells for improved anti-tumor efficacy against glioblastoma. Mol Ther.

[372] Donovan LK (2020). Locoregional delivery of CAR T cells to the cerebrospinal fluid for treatment of metastatic medulloblastoma and ependymoma. Nat Med.

[373] Pang L (2016). Exploiting macrophages as targeted carrier to guide nanoparticles into glioma. Oncotarget.

[374] Pang L, Zhu Y, Qin J, Zhao W, Wang J (2018). Primary M1 macrophages as multifunctional carrier combined with PLGA nanoparticle delivering anticancer drug for efficient glioma therapy. Drug Delivery.

[375] Ibarra LE (2020). Trojan horse monocyte-mediated delivery of conjugated polymer nanoparticles for improved photodynamic therapy of glioblastoma. Nanomedicine.

[376] Joice SL (2009). Modulation of blood–brain barrier permeability by neutrophils: in vitro and in vivo studies. Brain Res.

[377] Xue J (2017). Neutrophil-mediated anticancer drug delivery for suppression of postoperative malignant glioma recurrence. Nat Nanotechnol.

[378] Brackett CM, Muhitch JB, Evans SS, Gollnick SO (2013). IL-17 promotes neutrophil entry into tumor-draining lymph nodes following induction of sterile inflammation. J Immunol.

[379] Liu X (2021). Targeted photoacoustic imaging of brain tumor mediated by neutrophils engineered with lipid-based molecular probe. ACS Mater Lett.

[380] Wang GG, Calvo KR, Pasillas MP, Sykes DB, Häcker H, Kamps MP (2006). Quantitative production of macrophages or neutrophils ex vivo using conditional Hoxb8. Nat Methods.

[381] Hao J, Meng L-Q, Xu P-C, Chen M, Zhao M-H (2012). p38MAPK, ERK and PI3K signaling pathways are involved in C5a-primed neutrophils for ANCA-mediated activation. PLoS ONE.

[382] Wang J (2021). Inflammatory tumor microenvironment responsive neutrophil exosomes-based drug delivery system for targeted glioma therapy. Biomaterials.

[383] Zhang H (2021). Dual-responsive biohybrid neutrobots for active target delivery. Sci Robot.

[384] Hou J (2019). Accessing neuroinflammation sites: Monocyte/neutrophil-mediated drug delivery for cerebral ischemia. Sci Adv.

[385] D’Souza A, Dave KM, Stetler RA, Manickam DS (2021). Targeting the blood-brain barrier for the delivery of stroke therapies. Adv Drug Deliv Rev.

[386] Sun Y (2017). Advances of blood cell-based drug delivery systems. Eur J Pharm Sci.

[387] Magnani M, Rossi L (2014). Approaches to erythrocyte-mediated drug delivery. Expert Opin Drug Deliv.

[388] Pourgholi F, Hajivalili M, Farhad J-N, Kafil HS, Yousefi M (2015). Nanoparticles: novel vehicles in treatment of glioblastoma. Biomed Pharmacother.

[389] Pellegatta S (2006). Neurospheres enriched in cancer stem-like cells are highly effective in eliciting a dendritic cell-mediated immune response against malignant gliomas. Can Res.

[390] Chakroun RW, Zhang P, Lin R, Schiapparelli P, Quinones-Hinojosa A, Cui H (2018). Nanotherapeutic systems for local treatment of brain tumors. WIREs Nanomed Nanobiotechnol.

[391] Hersh AM, Alomari S, Tyler BM (2022). Crossing the blood-brain barrier: advances in nanoparticle technology for drug delivery in neuro-oncology. Int J Mol Sci.

